# Wogonin inhibits the proliferation of prolactinoma through the PI3K/AKT signaling pathway

**DOI:** 10.3389/fphar.2025.1546285

**Published:** 2025-05-15

**Authors:** Zhiyong Du, Cuiping Sun, Jiawei Wu, Hongwei Gao, Jialong Wu, You Zhou, Xuechao Wu, Liping Shen, Qing Wang

**Affiliations:** ^1^ Department of Neurosurgery, Wuxi No. 2 People’s Hospital (Jiangnan University Medical Center), Wuxi, China; ^2^ Wuxi School of Medicine, Jiangnan University, Wuxi, China; ^3^ Wuxi Neurosurgical Institute, Wuxi, China; ^4^ Neurological Medicine Research Centre, Jiangnan University, Wuxi, China

**Keywords:** network pharmacology, molecular docking, prolactinoma, wogonin, PI3K/Akt signaling pathway, apoptosis

## Abstract

**Objectives:**

This investigation sought to explore the inhibitory impact of wogonin on prolactinoma and elucidate its underlying mechanisms through network pharmacology, molecular docking (MD), and molecular biology experiments.

**Methods:**

Target identification for wogonin and prolactinoma was conducted using relevant databases, followed by protein-protein interaction (PPI) analysis of intersecting targets via the STRING database. Functional and pathway enrichment analyses were executed utilizing Gene Ontology (GO) and Kyoto Encyclopedia of Genes and Genomes (KEGG) methodologies. Hub genes were identified from the PPI network, and MD was utilized to assess the binding patterns and interaction strength between wogonin and hub targets. Network pharmacological findings were further validated through *in vivo* and *in vitro* experiments.

**Results:**

A sum of 137 drug targets for wogonin and 3,942 disease targets for prolactinoma were identified, with 37 overlapping targets. Nine hub genes were screened, including KDR, EGFR, BCL2, IL6, ESR1, MYC, CCL2, PTGS2, and ESR2. GO and KEGG analyses revealed that wogonin was closely associated with several critical signaling cascades. MD analysis confirmed robust binding interactions between wogonin and the identified hub targets. Cellular experiments suggested that wogonin suppressed cell proliferation and triggered apoptosis in prolactinoma cells in a time- and concentration-dependent manner, primarily via inhibition of the PI3K/AKT signaling cascades. Animal studies further revealed that wogonin markedly suppressed tumor growth and enhanced prolactinoma sensitivity to bromocriptine.

**Conclusion:**

These findings suggest that wogonin exerts its anti-prolactinoma effects via multiple targets and signaling cascades, establishing a robust scientific basis for the development and screening of novel anti-prolactinoma therapeutics.

## 1 Introduction

Prolactinoma, the most prevalent type of pituitary adenoma, accounts for approximately 40%–66% of functional pituitary adenomas (Tritos and Miller, 2023; Petersenn and Giustina, 2020). Although benign and not life-threatening, prolactinomas markedly impair patients’ quality of life due to clinical manifestations such as amenorrhea, galactorrhea, infertility, and visual disturbances (Castle-Kirszbaum et al., 2024; Auriemma et al., 2023). The primary treatment strategy involves dopamine agonists (Auriemma et al., 2023; Inder and Jang, 2022), however, many patients experience adverse effects or develop resistance (Maiter, 2019; Cheng et al., 2024; Vermeulen et al., 2020), undermining therapeutic efficacy and increasing recurrence risk (Fukuhara et al., 2022). Consequently, identifying novel therapeutic targets and drugs is critical to enhancing treatment outcomes and improving patient quality of life. Traditional Chinese medicines (TCM) serve as a valuable resource for new drug development due to their multi-component, multi-target characteristics, and low toxicity profiles (Wang et al., 2020; Liu Y. et al., 2020). Notably, approximately 52% of antineoplastic agents approved by the U.S. FDA are derived from natural products (Newman and Cragg, 2016). Wogonin (Wog), a flavonoid isolated from the Chinese herb Scutellaria baicalensis Georgi, demonstrates multiple biological functions, encompassing anti-inflammatory, antioxidant, anti-allergic, and antibacterial properties (Liao et al., 2021; Guo et al., 2022; Wang et al., 2021). Increasing evidence supports its antitumor efficacy across various malignancies (Banik et al., 2022; Zhang et al., 2022; Yang et al., 2020). For instance, wog promotes apoptosis in tumor cells by activating BAX proteins, with this effect in human prostate cancer cells linked to increased intracellular P53 expression (Lee et al., 2008). Furthermore, wog triggers apoptosis in lymphoma and breast cancer cells by suppressing anti-apoptotic protein levels, specifically Mcl-1, which belongs to the Bcl-2 family, and Survivin (Huang et al., 2012; Polier et al., 2015). Although its antitumor potential in prolactinoma has yet to be elucidated, wog’s established bioactivity underscores its value as a promising candidate for anticancer drug development.

Network pharmacology (NP) has emerged as an innovative discipline tailored to the multi-level and multi-target nature of TCM formulas (Zhao et al., 2023; Shang et al., 2023; Lin et al., 2023). It enables comprehensive analysis of drug-disease interactions from multiple perspectives, elucidating the mechanisms of action through gene functions and signaling pathways (Wu et al., 2024). This approach has gained recognition for its accuracy and reliability in exploring complex biological systems (Wang Y. X. et al., 2022; Liu Q. et al., 2023). In this study, the key molecular targets, biological processes, and signaling pathways underlying the effects of wog on prolactinoma were systematically identified and experimentally validated using a NP framework. These findings provide a robust theoretical and experimental foundation for future scientific investigations into the medicinal applications of wog.

## 2 Methods and materials

### 2.1 Chemicals and antibodies

Wogonin (W820521-50 mg, purity ≥99%) and bromocriptine (BRC) (B860726-50 mg, purity >99%) were acquired from Shanghai Macklin Biochemical Technology Co., Ltd. (Shanghai, China). Horse serum, fetal bovine serum, penicillin-streptomycin, and Ham’s F12K medium were supplied by Wuhan Procell Life Science and Technology Co., Ltd. (Wuhan, China). Primary antibodies, including anti-GAPDH (T0004), anti-PI3K (AF6242), anti-AKT (AF6261), and anti-Bax (AF0120), were obtained from Affinity Biosciences (Jiangsu, China). The anti-phospho-PI3K antibody (YP0224) was procured from ImmunoWay Biotechnology (Beijing, China), while anti-phospho-AKT (4060T), anti-Cleaved Caspase-3 (9661S), and anti-Cleaved Caspase-9 (9507S) were sourced from Cell Signaling Technology (Danvers, MA). A CCK-8 kit (MAO218) was obtained from Melone Pharmaceutical Co., Ltd. (Dalian, China), and an ELISA kit (RA20563) was supplied by Bioswamp (Wuhan, China). All reagents and antibodies were used in accordance with the manufacturers protocols.

### 2.2 Screening of wogonin drug targets

The Traditional Chinese Medicine Systems Pharmacology Database and Analysis Platform (TCMSP) was queried using the keyword “wogonin” to retrieve related targets, which were then matched to corresponding gene names via the UniProt database (https://www.uniprot.org). Unmatched genes were excluded. Additional drug targets were identified through searches of SwissTargetPrediction (http://swisstargetprediction.ch) and STITCH (http://stitch.embl.de) (Zhang et al., 2021; Li et al., 2023). After combining data from these sources and removing duplicates, the drug targets of wog were finalized.

### 2.3 Screening for prolactinoma disease targets

The GSE119063 dataset was procured through the Gene Expression Omnibus (GEO) database (https://www.ncbi.nlm.nih.gov/geo) (Wang Y. et al., 2022), and GEO2R was utilized to ascertain differentially expressed genes (DEGs) between prolactinoma tissues and normal pituitary tissues. DEGs were selected grounded in |logFC| > 1 and P < 0.05 thresholds (Zhang et al., 2023). Bioinformatics website (https://www.bioinformatics.com.cn) was utilized to generate volcano plots and heatmap (He et al., 2019). Prolactinoma-related disease targets were retrieved from the GeneCards (https://www.genecards.org) and PharmGKB (https://www.pharmgkb.org) databases utilizing the keyword “prolactinoma” with GeneCards results filtered by a relevance score >5 (Weng et al., 2023). Therapeutic targets for prolactinoma were compiled by merging these datasets and removing duplicates.

### 2.4 Identification of drug and disease common targets

Potential therapeutic targets for wog in prolactinoma were identified by intersecting drug targets and disease targets utilizing Venny 2.1.0 (https://bioinfogp.cnb.csic.es/tools/venny) (He et al., 2023).

### 2.5 Development of protein-protein interaction (PPI) network and identification of hub targets

The intersecting targets were analyzed in the STRING database (https://cn.string-db.org) to construct PPI networks (Li et al., 2022), which were subsequently visualized using Cytoscape software. Node parameters, encompassing betweenness unDir (BU), closeness unDir (CU), and degree unDir (DU), were computed utilizing the CentiScape plug-in (Liu H. et al., 2020). Targets with values exceeding threshold parameters for BU, CU, and DU were designated as hub targets for wog in prolactinoma therapy.

### 2.6 GO and KEGG enrichment analyses

The shared targets identified were analyzed using the DAVID database (https://david.ncifcrf.gov) for Gene Ontology (GO) enrichment and Kyoto Encyclopedia of Genes and Genomes (KEGG) pathway enrichment (Wang Y. et al., 2022; Luo et al., 2023). GO enrichment analysis categorized the targets into biological processes (BP), cellular components (CC), and molecular functions (MF), utilizing a significance threshold of P < 0.05. The top 10 enriched categories were visualized using bioinformatics website and presented as bubble charts (Madikyzy et al., 2022).

### 2.7 Molecular docking (MD) analysis

MD analysis of wog with hub targets was conducted using AutoDockTools-1.5.7 (He et al., 2023; Ji et al., 2023). The mol2 file of wog was retrieved from TCMSP, followed by hydrogen atom addition, ligand definition, and torsion bond selection. The processed ligand was saved in PDBQT format. Protein structures were procured from the PDB database (https://www.rcsb.org), preprocessed, and exported to PDBQT format. Docking parameters were configured, and docking simulations were performed using Autogrid and AutoDock. The structure exhibiting minimal binding energy was selected as the optimal result and rendered utilizing PyMOL (Ji et al., 2023; Lu et al., 2023).

### 2.8 Cell culture

Rat pituitary tumor cell lines MMQ and GH3 were procured from Wuhan Procell Life Science and Technology Co., Ltd. (Wuhan, China). The cells underwent cultivation in Ham’s F12K medium enriched with 15% horse serum, 2.5% fetal bovine serum, and 1% penicillin-streptomycin, sustained at 37°C within a 5% CO_2_ incubator (Selek et al., 2023).

### 2.9 Cell viability assay

The effects of wog on cell viability were assessed utilizing a CCK-8 kit. MMQ and GH3 cells were placed in 96-well plates at 1 × 10^4^ cells/well. Experimental groups included blank controls, untreated controls, and drug-treated groups, with five replicate wells per group. Outliers (maximum and minimum values) were excluded to minimize errors. After 24 h of initial culture, cells underwent exposure to multiple drug concentrations across 24, 48, and 72-hour periods. A volume of 10 µL CCK-8 solution was introduced into wells containing 100 µL medium, succeeded by incubation at 37°C for 3–4 h. The optical density measurements were procured at 450 nm utilizing a Multiskan FC microplate reader (Thermo Scientific, United States).

### 2.10 Colony formation assay

For colony formation assay, GH3 cells in the logarithmic growth phase were seeded in six-well plates at 1,000–2,000 cells/well, utilizing three replicates per group. After cell attachment (24 h), a fresh complete medium containing varying drug concentrations was introduced. Cells were kept at 37°C in a 5% CO_2_ atmosphere for 14–21 days, with medium replacement every 4–5 days. The resulting colonies were fixed, visualized with 0.1% crystal violet staining, imaged, and enumerated.

### 2.11 Enzyme-linked immunosorbent assay

To measure prolactin (PRL) secretion, MMQ and GH3 cells were placed in six-well plates at 1 × 10^5^ cells/well, utilizing three replicates per group. Cell supernatants were harvested after 72 h of drug treatment, and PRL levels were quantified utilizing a rat PRL ELISA kit per the supplier’s protocols. OD values were ascertained at 450 nm utilizing a Multiskan FC microplate reader (Thermo Scientific, United States). PRL concentrations in cell supernatants were calculated in ng/mL based on a standard curve generated with PRL standards provided in the kit.

### 2.12 Western blot analysis

GH3 and MMQ cells in the logarithmic growth phase were kept in Petri dishes and subjected to drug treatments for 72 h. Protein extraction was conducted utilizing RIPA lysis buffer comprising 1 mM enzyme and phosphatase blockers, with subsequent centrifugation executed at 12,000 rpm for 15 min under 4°C to isolate the supernatant. Protein levels were quantified utilizing a BCA assay kit, after which 5 × SDS-PAGE Loading Buffer was introduced, and the samples were denatured at 100°C for 10 min. The protein samples underwent SDS-PAGE separation, membrane transfer to PVDF, and BSA blocking for 1 h. Primary antibody incubation occurred overnight at 4°C, succeeded by secondary antibody application for 2 h at ambient temperature. An automated chemiluminescence imaging platform (Tanon-4600, Shanghai, China) enabled protein detection, while ImageJ software facilitated band intensity measurement. The experimental procedure was replicated independently five times.

### 2.13 Tumor cell xenograft nude mouse models

A xenograft model involving subcutaneous tumors was implemented using BALB/c nude mice to assess wog’s *in vivo* efficacy against prolactinoma. Female BALB/c nude mice (n = 20), aged 4–6 weeks, were obtained from SPF (Suzhou) Biotechnology Co., Ltd. and maintained in specific pathogen-free environments. All experimental procedures were conducted in compliance with the Regulations on the Management of Laboratory Animals and sanctioned by the Animal Ethics Committee of Jiangnan University (Ethics No. JN.No20240630b0240912 [371]). To develop the prolactinoma model, GH3 cells (roughly 1.0 × 10^7^) were introduced via subcutaneous injection into the mice’s lateral abdomen. Tumor dimensions were recorded every alternate day, with volume calculations following the formula: volume = 1/2 × length × width^2^. Upon tumors reaching 2–4 mm, the mice underwent random distribution into four groups: Control group, Wog group, BRC group and Wog + BRC group (*n* = 5). Mice in the Wog group received daily intragastric administration of wog (120 mg/kg/day i.g.); mice in the BRC group received daily intragastric administration of BRC (1 mg/kg/day i.g.); and the combined treatment group received daily administration of both BRC and wog. After 14 days of treatment, tumors were excised for measurement, weighing, and immunohistochemical analysis (Tang C. et al., 2021). To evaluate potential drug toxicity, internal organs (heart, liver, spleen, lungs, and kidneys) and blood samples were procured for histopathological examination via HE staining and serum biochemical analysis.

### 2.14 Immunohistochemistry

Immunohistochemical staining was executed on paraffin-embedded tumor tissue sections to evaluate target protein expression. Following dewaxing and hydration, the slides were treated with citric acid antigen retrieval solution and antigen restoration process. Non-specific binding was prevented by treating the slides with 10% BSA for 30 min at ambient temperature, and the excess solution was gently removed. The slides were then exposed to the primary antibody overnight at 4°C, succeeded by exposed to horseradish peroxidase-conjugated secondary antibody for 20 min at ambient temperature. Nuclear staining was conducted with hematoxylin to visualize nuclei. Images of stained sections were captured using a microscope.

### 2.15 Hematoxylin-eosin (HE)

For HE staining, tissue samples were paraffin-embedded and sectioned following dehydration and clearing. Deparaffinization was performed with xylene and ethanol, after which the specimens were stained with hematoxylin for 5 min. Following differentiation, the specimens were counterstained with 0.5% eosin for 1 min, rinsed with water, and exposed to ethanol dehydration and xylene clearing. The specimens were sealed with neutral gum and examined microscopically, with representative images captured.

### 2.16 Statistical analysis

Experimental data underwent statistical analysis utilizing GraphPad Prism version 9.0. Each experiment was performed in triplicate or more, with outcomes presented as mean ± standard deviation (SD). Group comparisons were made utilizing one-way ANOVA, with statistical significance established at *p < 0.05* (*p < 0.05, **p < 0.01, ***p < 0.005, ****p < 0.0001; ns means no significance).

## 3 Results

### 3.1 Identification of gene targets for wogonin in prolactinoma

**FIGURE 1:**
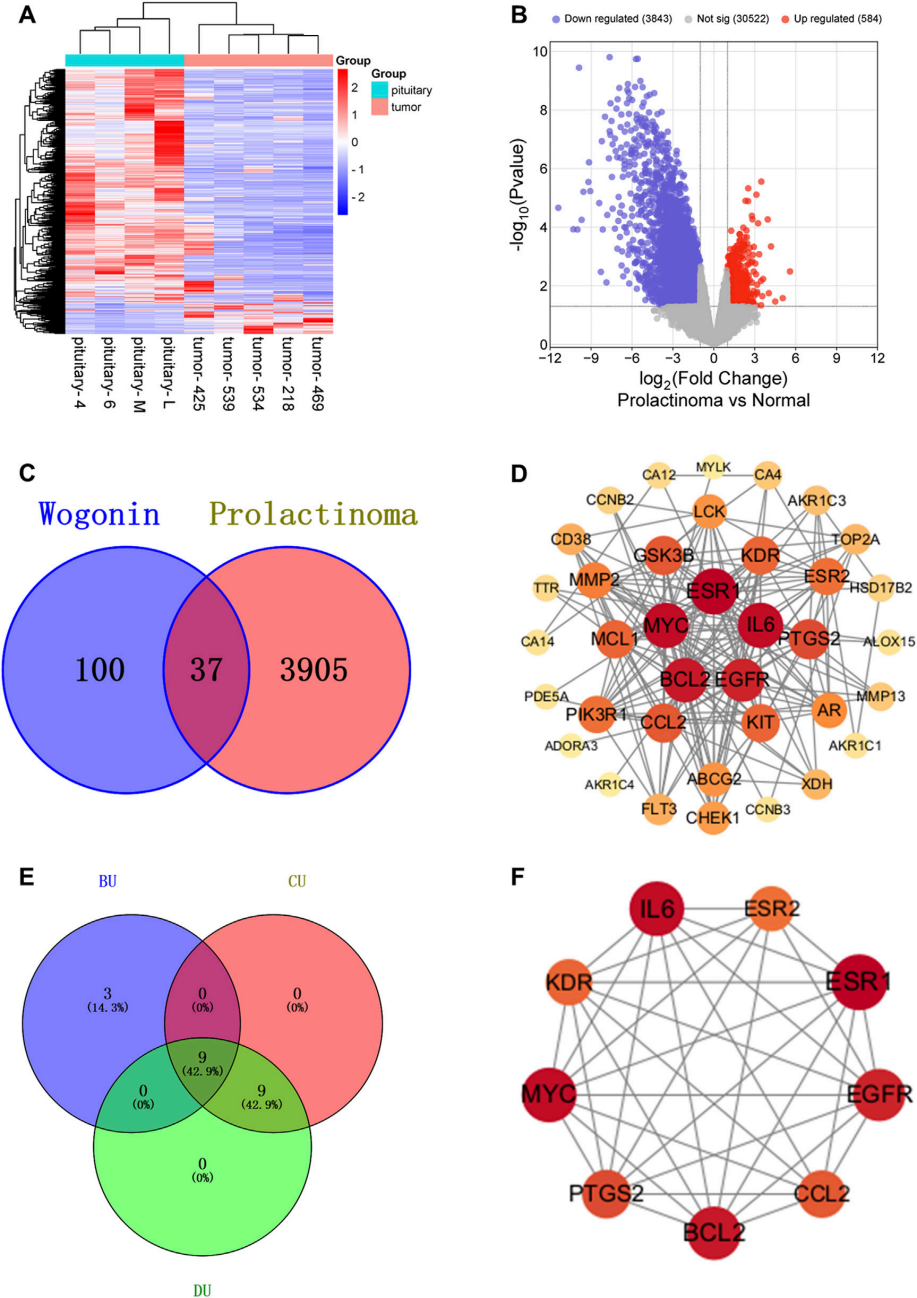
Relevant targets, PPI network and hub targets: **(A)**. Volcano plot of prolactinoma DEGs in the GEO database **(B)**. Heatmap of prolactinoma DEGs in the GEO database **(C)**. Venn diagram showing the common part of wogonin and prolactinoma **(D)**. PPI network of potential targets for wogonin therapy of prolactinoma **(E,F)**. Hub targets for wogonin therapy of prolactinoma.

A total of 35, 103, and 10 pharmacological targets of wog were identified through the TCMSP, SwissTargetPrediction, and STITCH databases, respectively. After eliminating duplicates and erroneous entries, 137 unique pharmacological targets were finalized. Differential expression analysis of the GSE119063 dataset from the GEO database identified 3,909 DEGs associated with prolactinoma (Figures 1A,B). Additionally, 46 disease-related genes were procured from the GeneCards database and 8 from PharmGKB. Integrating these datasets and removing redundancies resulted in 3,942 prolactinoma-associated targets. By intersecting the pharmacological and disease target datasets, 37 overlapping genes were identified (Figure 1C).

### 3.2 Development of PPI network and detection of hub targets

The intersecting genes were analyzed for PPI relationships utilizing the STRING database, and the generated PPI network was rendered via the Cytoscape tool. The network comprised 37 nodes and 180 edges (Figure 1D), with node size and color intensity proportional to their degree values. Using the CentiScape plugin, hub targets were ascertained grounded in criteria for BU > 37.29729729729731, CU > 0.0142901334866273, and DU > 9.72972972972973 (Figures 1E,F). This analysis highlighted 9 hub targets: KDR, EGFR, BCL2, IL6, ESR1, MYC, CCL2, PTGS2, and ESR2 (Table 1).

**TABLE 1:** 9 hub targets identified using CentiScape plugin.

Gene symbol	Full name	Betweenness unDir	Closeness unDir	Degree unDir
BCL2	B-cell lymphoma-2	45.70052932	0.018181818	21
CCL2	Chemokine (C-C motif) ligand 2	104.9236037	0.016666667	16
EGFR	Epidermal growth factor receptor	62.19398989	0.018181818	20
ESR1	Estrogen receptor 1	285.3271758	0.020408163	23
ESR2	Estrogen receptors beta	58.00522876	0.016393443	14
IL6	Interleukin 6	101.4970088	0.018518519	22
KDR	Kinase insert domain receptor	40.2766082	0.016666667	15
MYC	Myelocytomatosis oncogene	165.0447178	0.018867925	22
PTGS2	Prostaglandin-endoperoxide synthase 2	86.46329819	0.01754386	17

### 3.3 GO and KEGG pathway enrichment analyses

**FIGURE 2:**
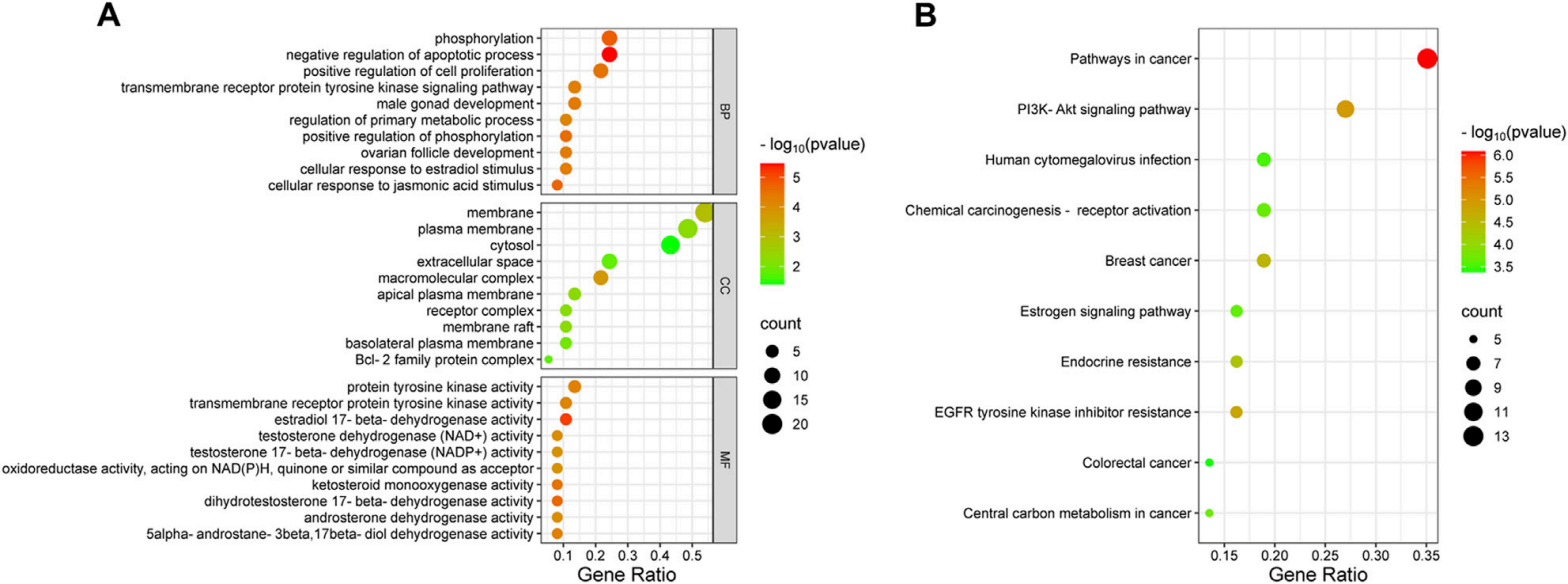
Bubble plot of enrichment analysis: **(A)**. GO functional enrichment analysis of wogonin in prolactinoma **(B)**. KEGG pathway enrichment analysis of wogonin in prolactinoma.

Functional enrichment analysis identified 142 BP, 14 CC, 52 MF terms, and 69 signaling pathways markedly associated with P < 0.05. The top 10 terms from GO and KEGG pathway enrichment were depicted in bubble plots. Key BP terms included negative regulation of apoptotic processes, phosphorylation, cellular response to jasmonic acid, and the transmembrane receptor protein tyrosine kinase signaling pathway. CC terms were primarily related to macromolecular complexes, membranes, apical plasma membranes, membrane rafts, and receptor complexes. MF terms were enriched in activities such as estradiol 17-beta-dehydrogenase, dihydrotestosterone 17-beta-dehydrogenase, and transmembrane receptor protein tyrosine kinase (Figure 2A). KEGG pathway examination suggested substantial enrichment in pathways encompassing pathways in cancer, PI3K/Akt signaling, EGFR tyrosine kinase inhibitor resistance, endocrine resistance, and central carbon metabolism in cancer (Figure 2B). Among these, the PI3K/Akt signaling cascade was notably associated with cancer progression, highlighting its critical function in tumor cell proliferation, apoptosis, and invasion (He et al., 2021; Dworakowska et al., 2009). These observations suggest that wog may exert therapeutic effects in prolactinoma through modulation of the PI3K/Akt signaling cascade.

### 3.4 Molecular docking

**FIGURE 3:**
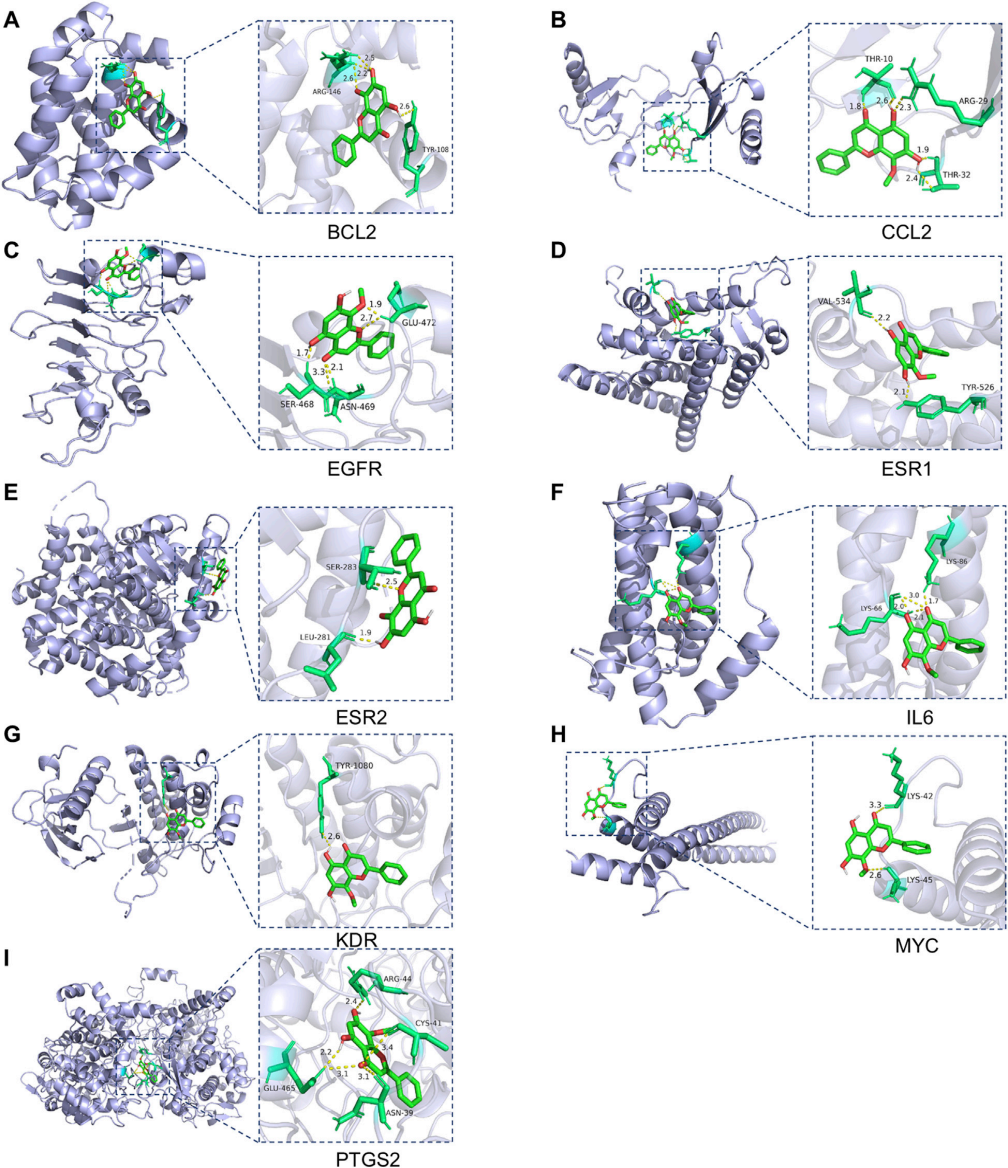
Molecular docking pattern of wogonin and hub target proteins: **(A)**. Wogonin-BCL2, **(B)**. Wogonin-CCL2, **(C)**. Wogonin-EGFR, **(D)**. Wogonin-ESR1, **(E)**. Wogonin-ESR2, **(F)**. Wogonin-IL6, **(G)**. Wogonin-KDR, **(H)**. Wogonin-MYC, **(I)**. Wogonin-PTGS2.

MD analysis was conducted to assess the interactions between wog and the identified hub targets, thereby verifying the reliability of these interactions. A lower binding energy between a small molecule ligand and a protein receptor indicates stronger and more stable affinity. The MD results showed that the binding energies between wog and the target proteins ranged from −3.83 to −6.67 kcal/mol (Table 2). Wog is tightly linked to amino acid residues through hydrogen bonds (Figure 3). The analysis demonstrated robust binding affinities between wog and the hub targets, supporting its potential therapeutic efficacy in prolactinoma treatment.

**TABLE 2:** Molecular docking binding energies.

Core target	PDB ID	Binding energy (kcal/Mol)	Residue involved in H bonding
BCL2	6GL8	−4.18	ARG-146; TYR-108
CCL2	1DOK	−5.63	THR-10; ARG-29; THR-32
EGFR	3P0Y	−6.67	GLU-472; SER-468; ASN-469
ESR1	1XPC	−6.38	VAL-534; TYR-526
ESR2	3OLL	−5.19	SER-283; LEU-281
IL6	1ALU	−4.36	LYS-66; LYS-86
KDR	1YWN	−4.83	TYR-1080
MYC	5I4Z	−3.83	LYS-42; LYS-45
PTGS2	5FDQ	−5.58	ARG-44; CYS-41; ASN-39; GLU-465

### 3.5 Wogonin inhibits the prolactinoma cell proliferation

**FIGURE 4:**
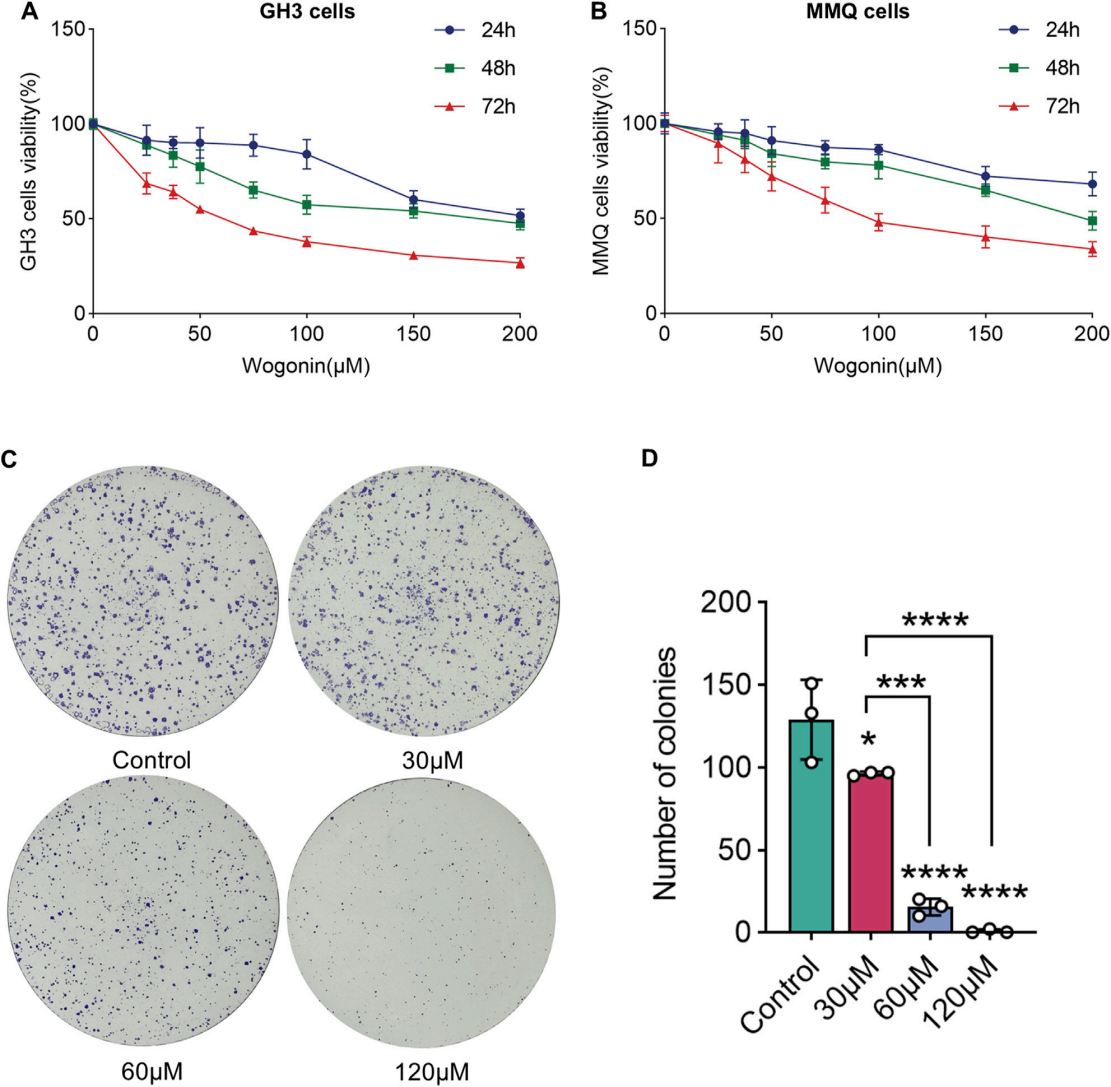
Effects of wogonin on cell viability and colony formation: GH3 cell **(A)** and MMQ cell **(B)** survival was determined by the CCK-8 assay **(C)**. GH3 cells were treated with wogonin at concentrations of 0, 30, 60 and 120 μM **(D)**. The statistical graph of the result of the colony formation. (****p < 0.0001, ***p < 0.005, **p < 0.01, *p < 0.05, Except for * on the n-zig-zag line, all other * indicate comparisons with the control group).

To examine how wog influences prolactinoma cell growth, GH3 and MMQ cells underwent treatment with diverse wog doses (0, 25, 37.5, 50, 75, 100, 150 and 200 μM) for 24, 48, and 72 h. Wog suppressed prolactinoma cell proliferation in a time- and concentration-dependent manner (Figures 4A,B). The calculated IC50 values for GH3 and MMQ cells were 61.99 μM and 107 μM, respectively, after 72 h of treatment.

### 3.6 Wogonin inhibits the colony formation ability of prolactinoma cell

Further experiments were executed to evaluate wog’s influence on colony formation. GH3 cells were exposed to 30 μM, 60 μM, and 120 μM wog, respectively. A significant reduction in colony formation was observed when treated with wog at concentrations of 30 μM and 60 μM. When the concentration of wog was 120 μM, colony formation was nearly completely inhibited (Figures 4C,D). These results indicate that wog effectively suppresses the colony formation ability of prolactinoma cells *in vitro*.

### 3.7 Wogonin inhibits PI3K/AKT pathway phosphorylation and induces apoptosis in prolactinoma cells

**FIGURE 5:**
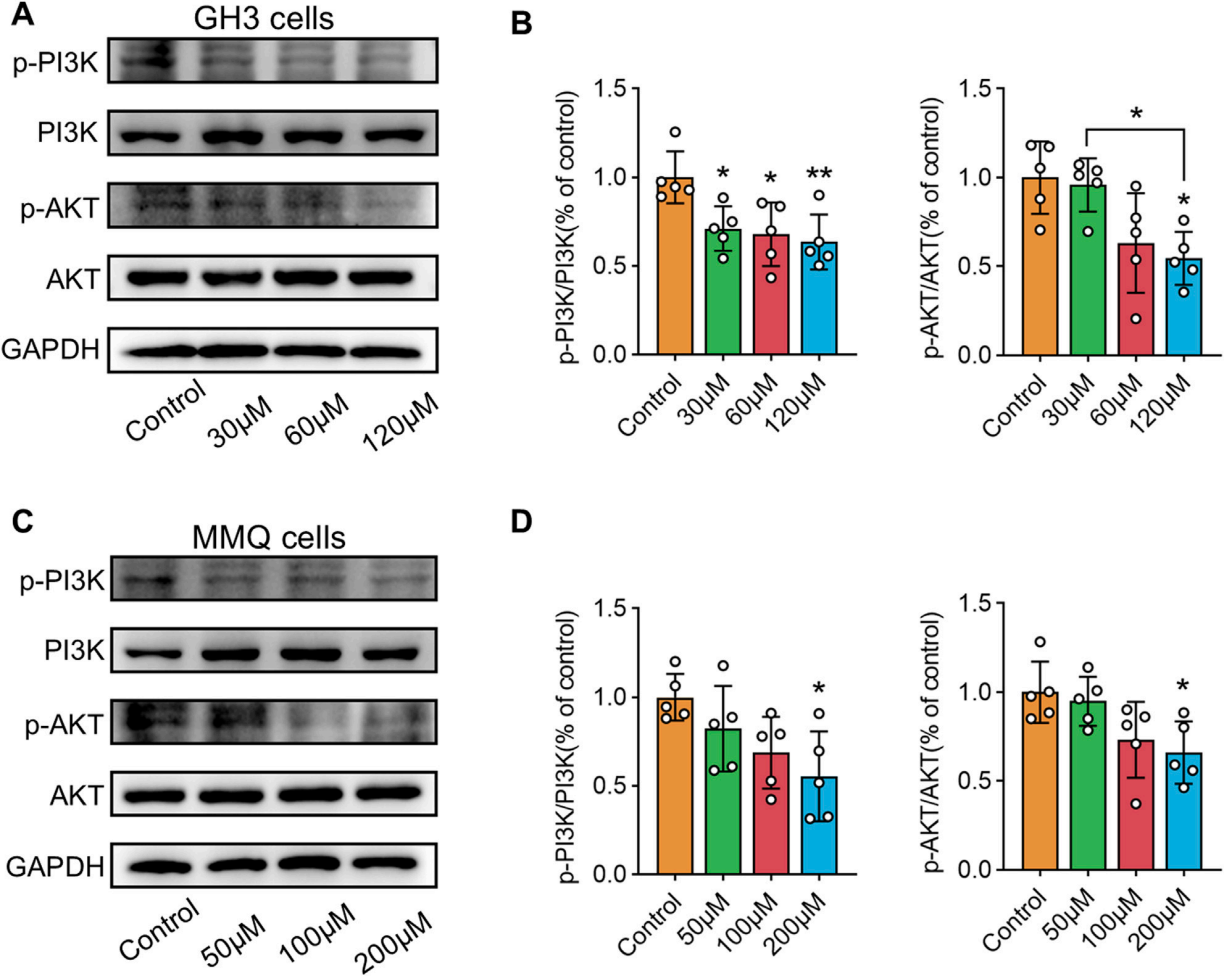
Protein expression levels of p-PI3K and p-AKT expression in prolactinoma cells were detected by Western blot. GAPDH was used as an internal control. n = 5/group: **(A)**. GH3 cells were treated with wogonin at concentrations of 0, 30, 60 and 120 μM for 72 h **(B)**. Statistical graph of Western blot results of GH3 cells **(C)**. MMQ cells were treated with wogonin at concentrations of 0, 50, 100 and 200 μM for 72 h **(D)**. Statistical graph of Western blot results of MMQ cells. (**p < 0.01, *p < 0.05. Except for * on the n-zig-zag line, all other * indicate comparisons with the control group).

**FIGURE 6:**
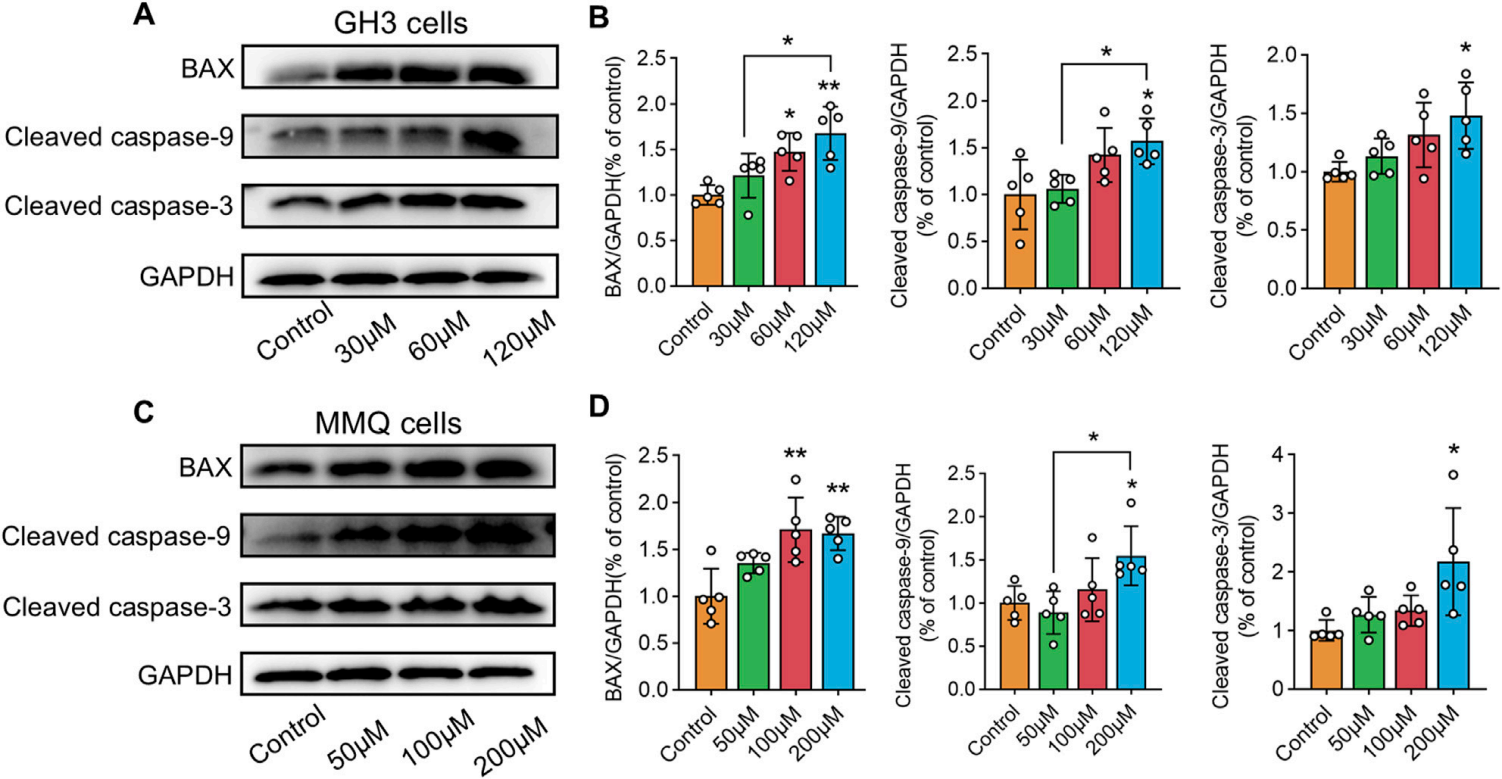
Protein expression levels of Bax, cleaved caspase-9 and cleaved caspase-3 expression in prolactinoma cells were detected by Western blot. GAPDH was used as an internal control. n = 5/group: **(A)**. GH3 cells were treated with wogonin at concentrations of 0, 30, 60 and 120 μM for 72 h **(B)**. Statistical graph of Western blot results of GH3 cells **(C)**. MMQ cells were treated with wogonin at concentrations of 0, 50, 100 and 200 μM for 72 h **(D)**. Statistical graph of Western blot results of MMQ cells. (**p < 0.01, *p < 0.05, Except for * on the n-zig-zag line, all other * indicate comparisons with the control group).

NP analysis indicated that wog’s tumor-suppressing activity potentially operates through PI3K/AKT pathway modulation. To test this hypothesis, GH3 and MMQ cells were exposed to diverse concentrations of wog for 72 h, and the PI3K/AKT pathway protein level was examined by Western blotting. Compared to the control group, wog markedly reduced phosphorylated PI3K (p-PI3K) and phosphorylated AKT (p-AKT) levels in GH3 cell (Figures 5A,B) and MMQ cell (Figures 5C,D). Given prior evidence that wog induces apoptosis in various tumor cells, its apoptotic effect on prolactinoma cells was also examined. Western blot analysis suggested that treatment with wog notably elevated pro-apoptotic protein levels in GH3 cell (Figures 6A,B) and MMQ cell (Figures 6C,D), encompassing Bax, cleaved caspase-9, and cleaved caspase-3. These results collectively indicate that wog inhibits the PI3K/AKT signaling pathway and promotes apoptosis in prolactinoma cells.

### 3.8 Wogonin inhibits PRL secretion in prolactinoma cells

**FIGURE 7:**
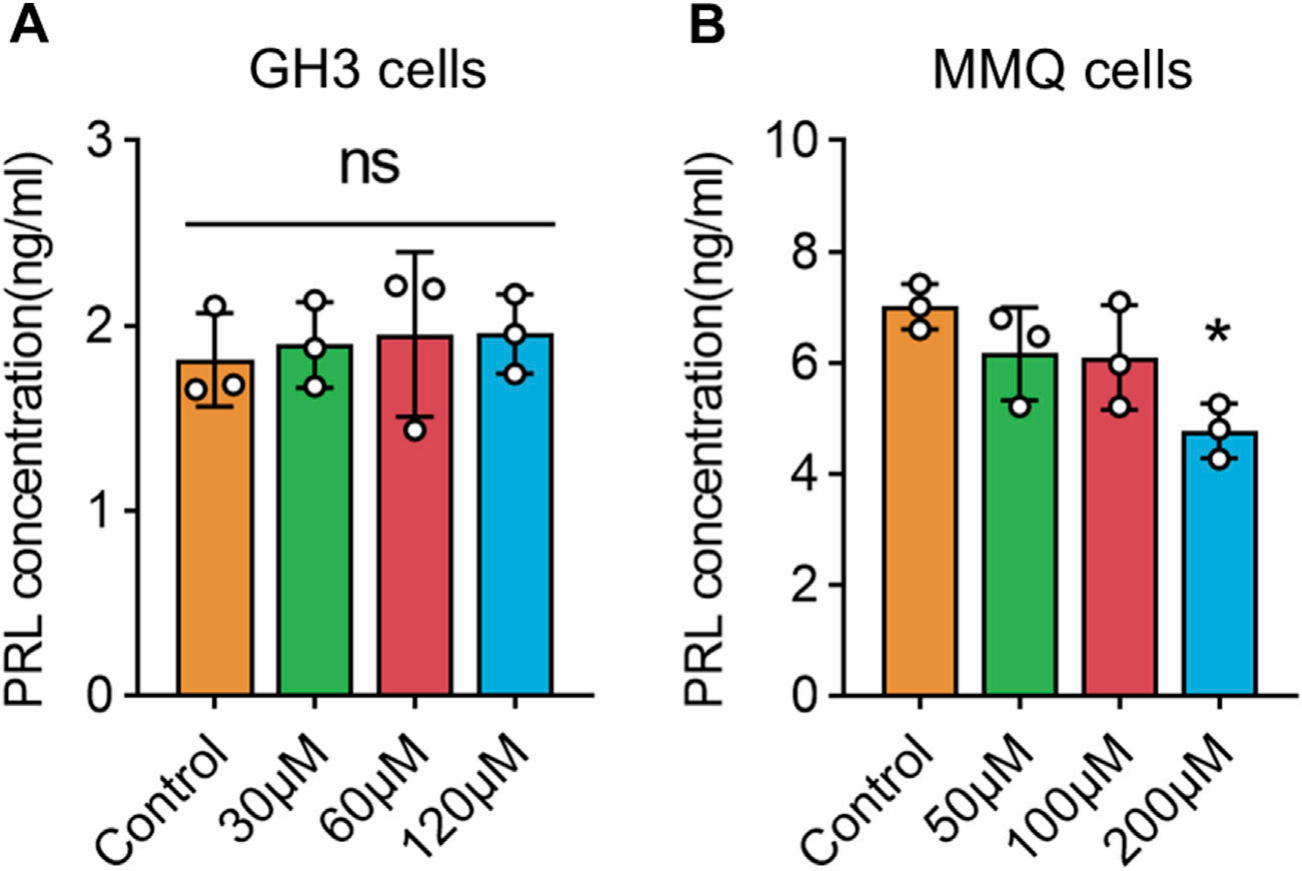
Levels of PRL secretion under wogonin treatment in prolactinoma cells: **(A)**. GH3 cells were treated with wogonin at concentrations of 0, 30, 60 and 120 μM for 72 h **(B)**. MMQ cells were treated with wogonin at concentrations of 0, 50, 100 and 200 μM for 72 h (*p < 0.05, all * indicate comparisons with the control group, ns means no significance).

PRL secretion levels in cell supernatants were measured using an ELISA kit following 72 h of wog treatment at various concentrations. In GH3 cells, wog treatment did not affect PRL secretion (Figure 7A). However, in MMQ cells, a marked decline in PRL levels was evident at 200 μM relative to the control group (Figure 7B). At lower concentrations (50 μM and 100 μM), no significant changes in PRL secretion were detected.

### 3.9 Wogonin inhibits prolactinoma cell growth *in vivo*

**FIGURE 8:**
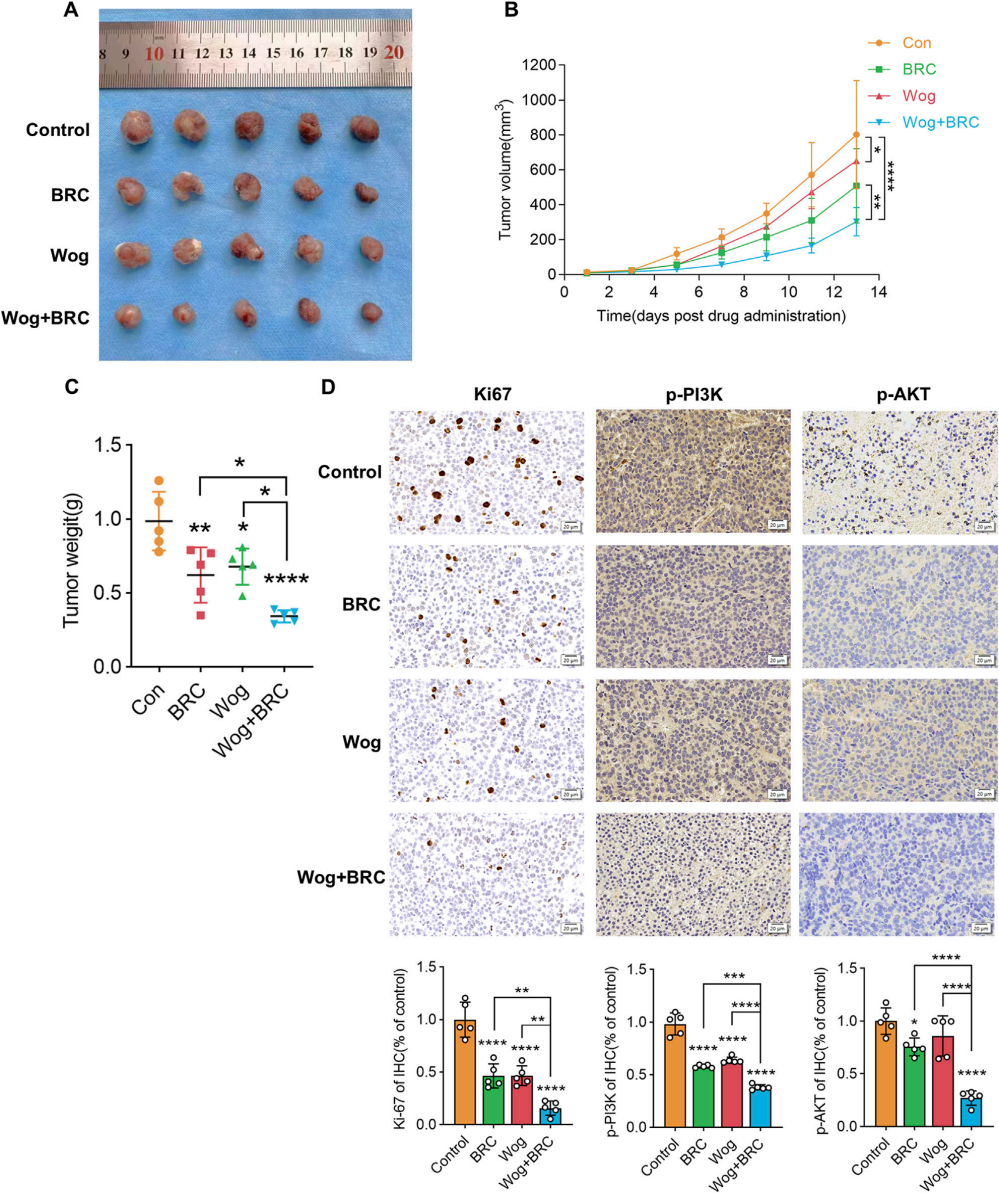
Subcutaneous xenograft model and drug treatment *in vivo*: **(A)** Representative images of xenograft tumors in nude mice **(B,C)**. After tumor formation, the tumor volume **(B)** and weight **(C)** of each group were assessed **(D)**. The immunohistochemical analyses of Ki-67, p-PI3K and p-AKT in tumor samples of each group. (****p < 0.0001, ***p < 0.005, **p < 0.01, *p < 0.05. Except for * on the n-zig-zag line, all other * indicate comparisons with the control group).

**FIGURE 9:**
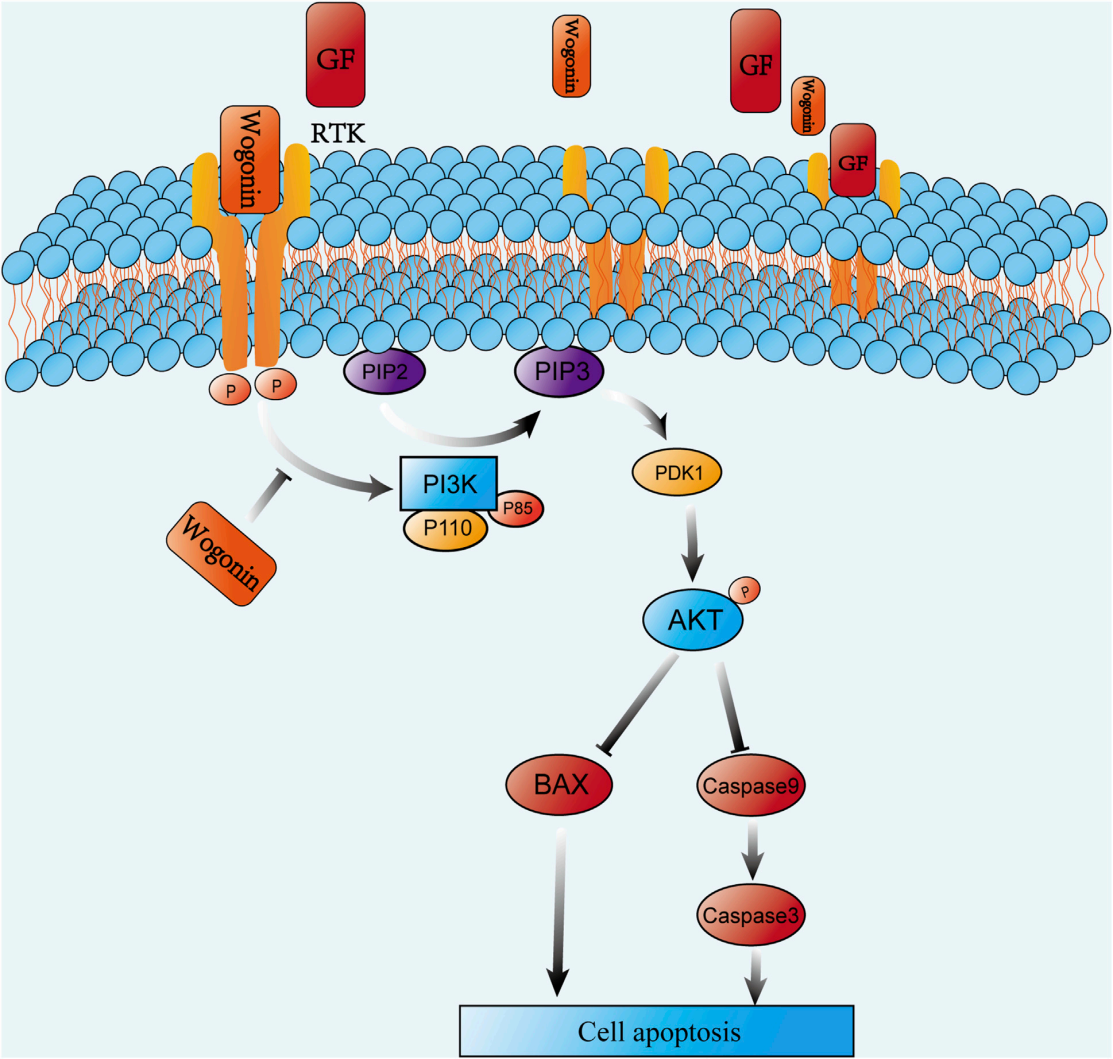
Proposed mechanism of wogonin-induced apoptosis in prolactinoma cells.

To examine how wog impacts prolactinoma development *in vivo*, a subcutaneous tumor xenograft model was developed using nude mice implanted with rat pituitary tumor cells. Following tumor formation, the mice were separated into four equal groups (n = 5 per group). Wog treatment markedly suppressed tumor growth (Figure 8A), with tumor size and weight in the Wog + BRC group markedly diminished relative to the control group or the single-drug treatment groups (Figures 8B,C). Immunohistochemical analysis revealed markedly reduced Ki-67, p-PI3K, and p-AKT levels in tumors treated with wog and BRC (Figure 8D). The combination therapy further enhanced the suppression of these proteins compared to individual treatments. These results demonstrate that wog inhibits prolactinoma cell proliferation and has a synergistic therapeutic effect with BRC, primarily through inhibition of the PI3K/AKT signaling pathway. Drug toxicity assessments, including HE staining of major organs, revealed no significant pathological changes in the treated groups (Supplementary Figure S2). Serum biochemical analyses confirmed that wog did not cause notable damage to liver or kidney function or to the myocardium (Supplementary Figure S1), suggesting a favorable safety profile for wog at the administered doses. Taken together, these results suggest that wog can induce apoptosis in prolactinoma cells by inhibiting phosphorylation of the PI3K/AKT pathway (Figure 9).

## 4 Discussion

BRC, the first-line clinical treatment for prolactinoma (Inder and Jang, 2022), suppresses prolactin secretion through negative feedback by activating dopamine receptors in the anterior pituitary. While this treatment effectively reduces prolactin levels and tumor size in the majority of patients, approximately 20% exhibit resistance to BRC (Maiter, 2019; Molitch, 2005). For these cases, temozolomide has demonstrated some efficacy as an adjunct to surgical and radiotherapeutic interventions (Tang H. et al., 2021). However, no definitive consensus exists regarding the optimal therapeutic approach, underscoring the urgent need for alternative strategies to manage dopamine-resistant prolactinomas.

The use of TCM in oncology has a longstanding history, with Scutellaria baicalensis Georgi being a prominent example. One of its primary bioactive constituents, wog, exhibits potent antitumor properties through various mechanisms (Huynh et al., 2017; Liu et al., 2024; Liu X. et al., 2023), encompassing apoptosis induction, angiogenesis inhibition, cell cycle arrest, suppression of tumor invasion and metastasis, and regulation of telomerase activity. This study employed a systematic approach to identify the potential molecular targets of wog for prolactinoma treatment, incorporating multiple analytical perspectives. Nine hub targets were subsequently identified, and MD analysis confirmed strong binding affinities between wog and these targets. These findings highlight wog’s potential as a promising therapeutic agent against prolactinoma, offering mechanistic insights into its antitumor efficacy.

GO and KEGG enrichment analyses revealed that wog exerts therapeutic effects on prolactinomas through various biological mechanisms. Key signaling pathways enriched in the analysis include Pathways in cancer, PI3K/Akt signaling, EGFR tyrosine kinase inhibitor resistance, endocrine resistance, and central carbon metabolism in cancer. Among these, the PI3K/AKT pathway emerges as a critical intracellular signaling cascade intricately linked to cellular growth, proliferation, metabolism, and tumorigenesis (He et al., 2021; Fresno et al., 2004). This pathway is regulated by upstream tyrosine kinase receptors and modulated by PI3K. Aberrant stimulation of the PI3K/AKT signaling cascade has been extensively reported as a driver of tumor progression, poor prognosis, and the emergence of therapeutic resistance (Wei et al., 2019; Song et al., 2020; Tufail et al., 2024). In prolactinoma, activation of the PI3K/Akt pathway is closely associated with cell proliferation (Ferraris, 2022). As a result, this pathway has become a prominent target in cancer therapy, with several small-molecule inhibitors such as Alpelisib, a PI3K inhibitor, and Capivasertib, a selective AKT inhibitor, showing promising anti-tumor efficacy in clinical studies, particularly for cancers harboring PI3K or AKT mutations (Savas et al., 2022; Turner et al., 2023).

The results of NP analyses were further validated through both *in vitro* and *in vivo* experiments. The results of cellular experiments showed that inhibited the phosphorylation of the PI3K/AKT pathway and induced apoptosis in prolactinoma cells. The results showed that GH3 cells and MMQ cells had different sensitivities to wog, the reason for which may be related to the different receptors on the surface of the 2 cells and the degree of activation of intracellular signaling pathways. In *in vivo* experiments, wog inhibited proliferation of prolactinoma. When combined with BRC, it demonstrated synergistic therapeutic effects on prolactinoma. However, the study has several limitations. First, the drug and disease targets were sourced from public databases such as TCMSP and GEO, which may contain incomplete or outdated data due to inconsistent update frequencies. Second, the experimental focus was limited to the molecular biology of the PI3K/AKT pathway, without exploring other potential mechanisms in depth. Third, the study relied on rat prolactinoma cells, which may not fully reflect the physiological and pathological processes in humans. Subsequent investigations ought to broaden the experimental parameters to encompass human-derived cells and explore additional pathways to improve the reliability, clinical relevance, and translational potential of the findings.

## 5 Conclusion

In summary, wog effectively inhibits the proliferation of prolactinoma cells and promotes tumor cell apoptosis in a time- and concentration-dependent manner. Furthermore, its antitumor efficacy is markedly enhanced when used in combination with BRC. These results underscore wog’s potential as a promising, safe, and effective therapeutic agent for prolactinoma, offering a novel avenue for future drug development and clinical treatment strategies.

## Data Availability

The datasets presented in this study can be found in online repositories. The names of the repository/repositories and accession number(s) can be found below: Du, zhiyong (2025), “Wogonin”, Mendeley Data, v2, doi: 10.17632/jtyrvxgwwv.2, https://data.mendeley.com/datasets/jtyrvxgwwv/2.
