# Supplementary material for: Wogonin inhibits the proliferation of prolactinoma through the PI3K/AKT signaling pathway
Source: Front Pharmacol. 2025 May 15;16:1546285. doi: 10.3389/fphar.2025.1546285 (PMC12119628; doi:10.3389/fphar.2025.1546285)
Supplement: Supplementary file 1 [file DataSheet2.docx]

***Supplementary Material***

Raw data of Western Blot


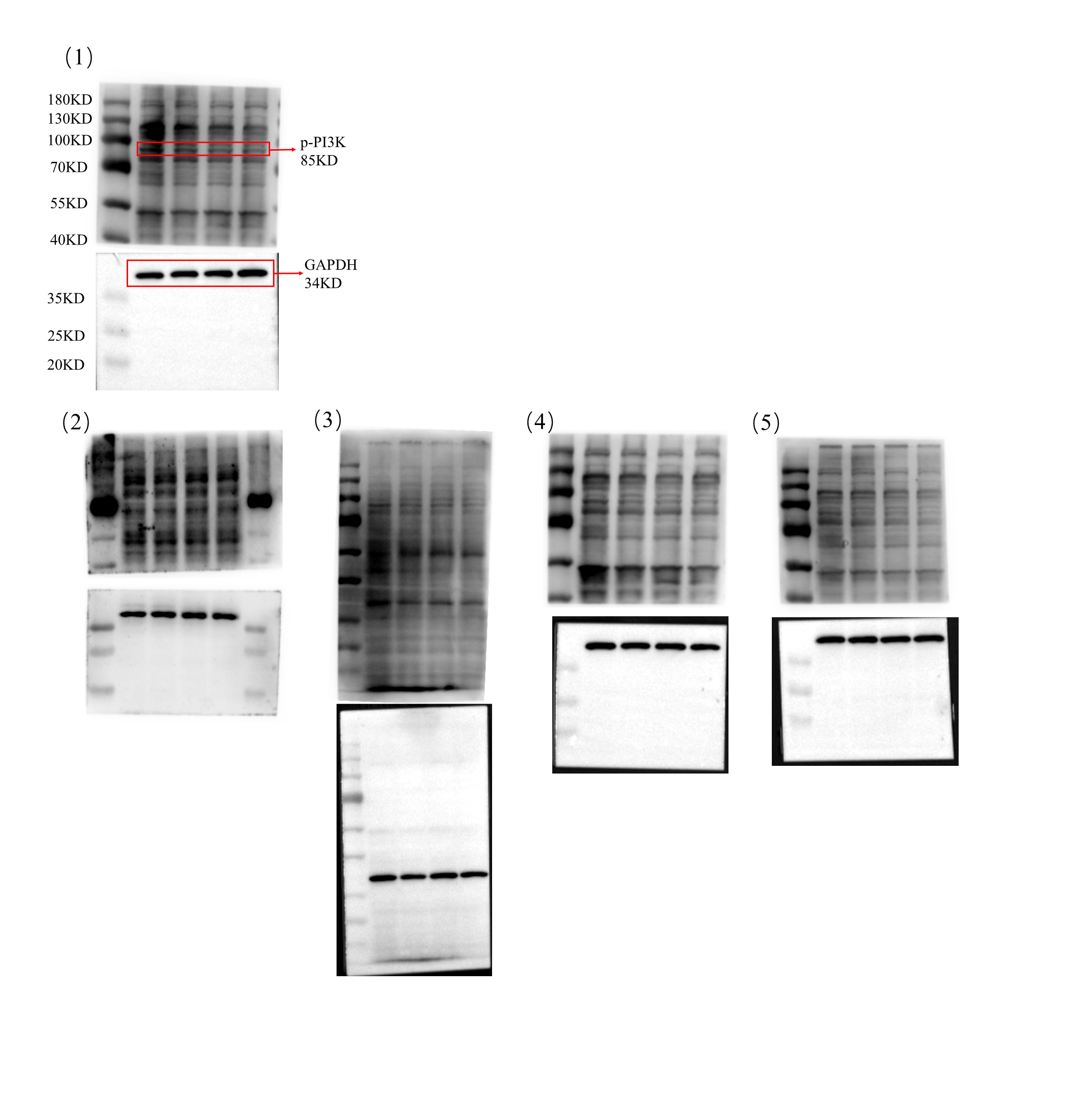


Expression of p-PI3K in GH3 cells, 5 replicate experiments.


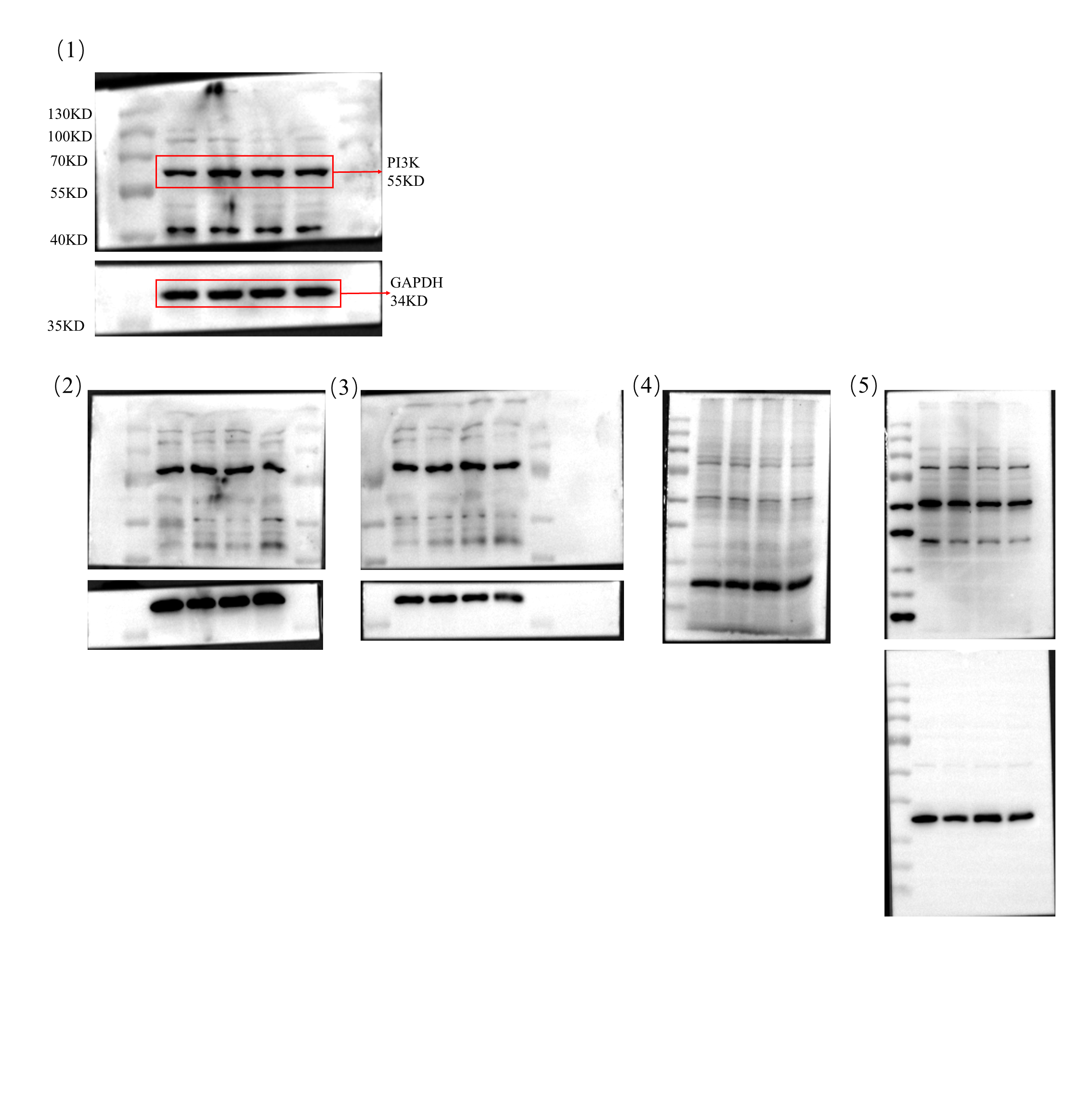


Expression of PI3K in GH3 cells, 5 replicate experiments.


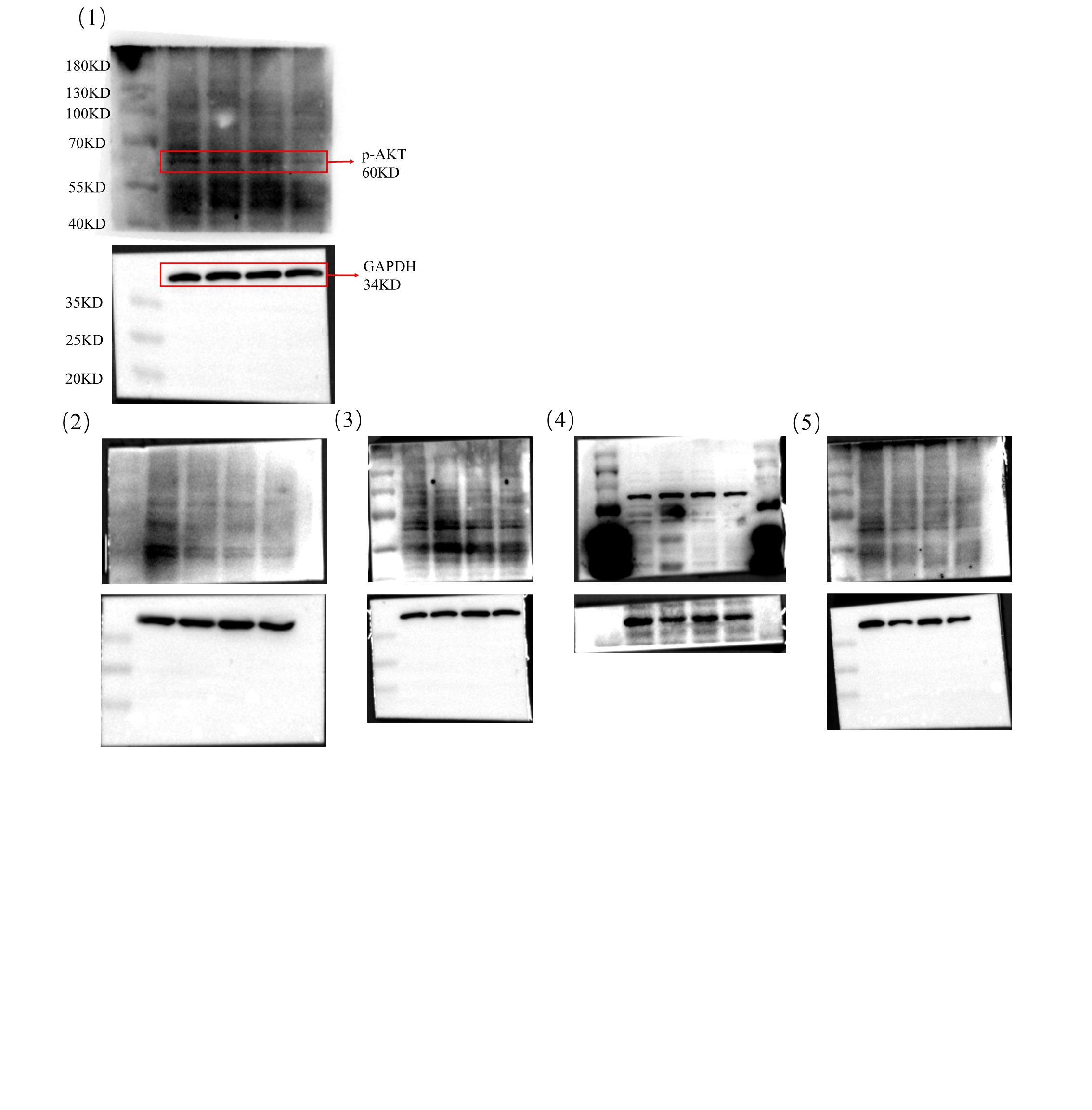


Expression of p-AKT in GH3 cells, 5 replicate experiments.


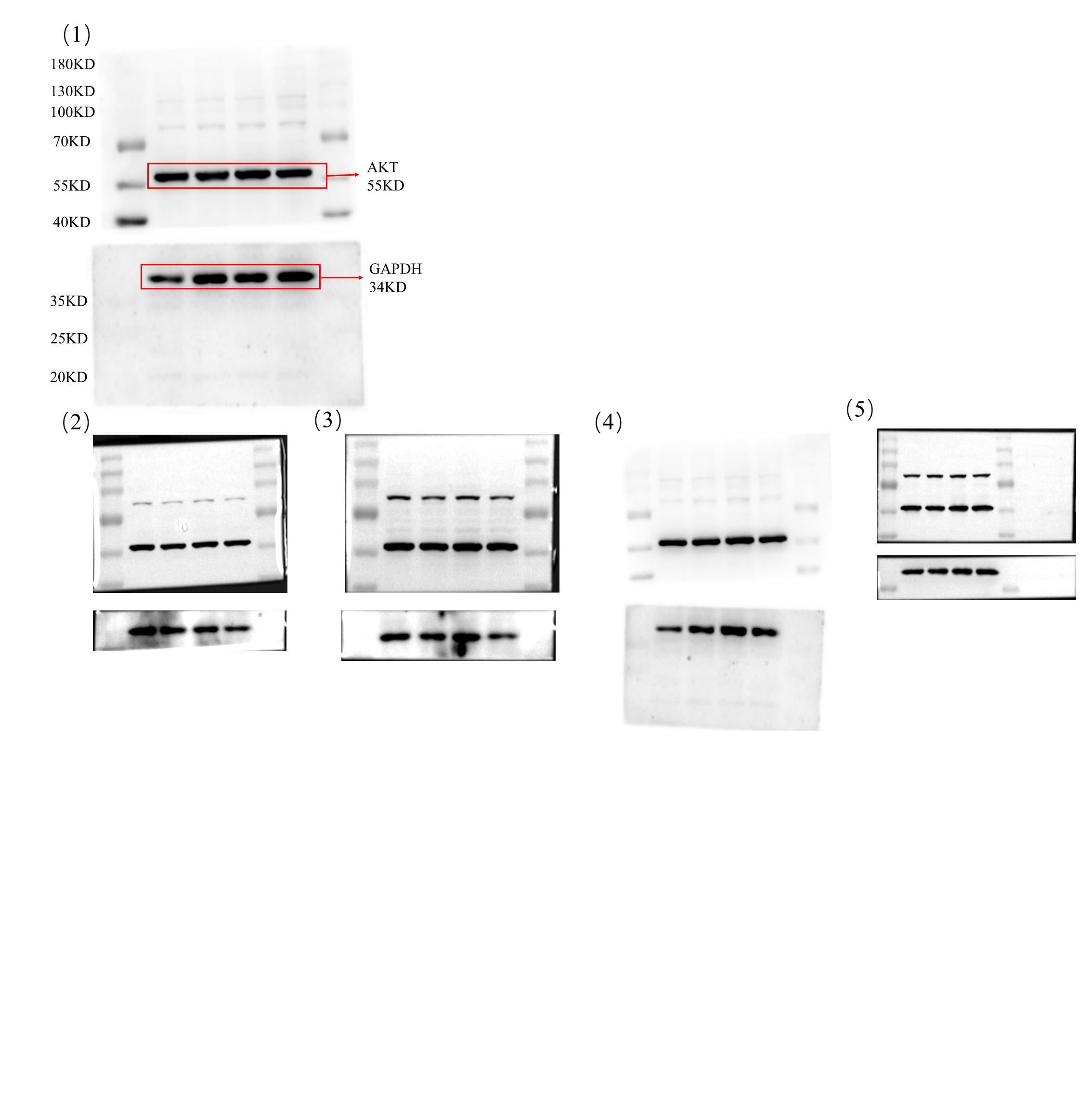


Expression of AKT in GH3 cells, 5 replicate experiments.


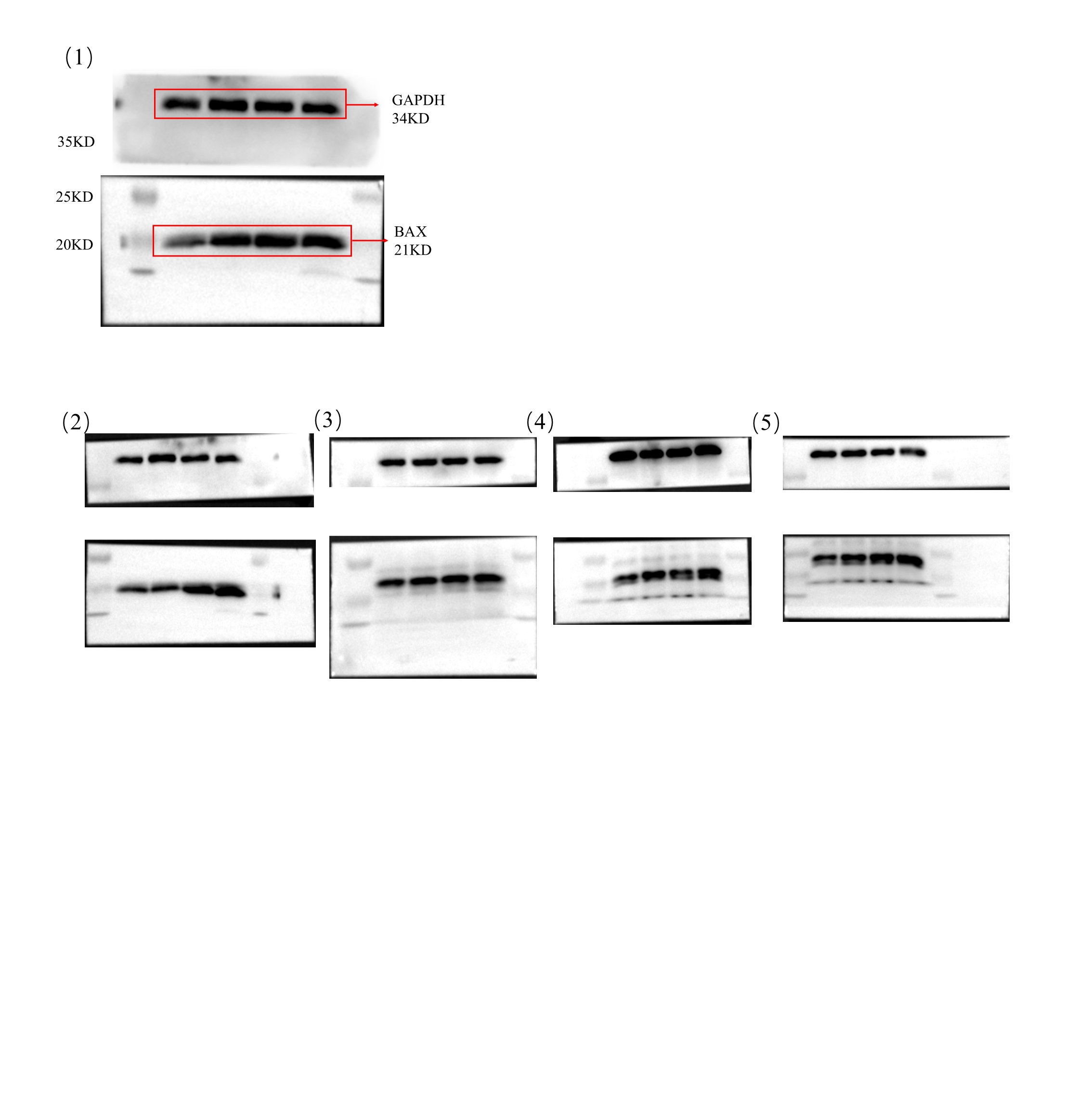


Expression of BAX in GH3 cells, 5 replicate experiments.


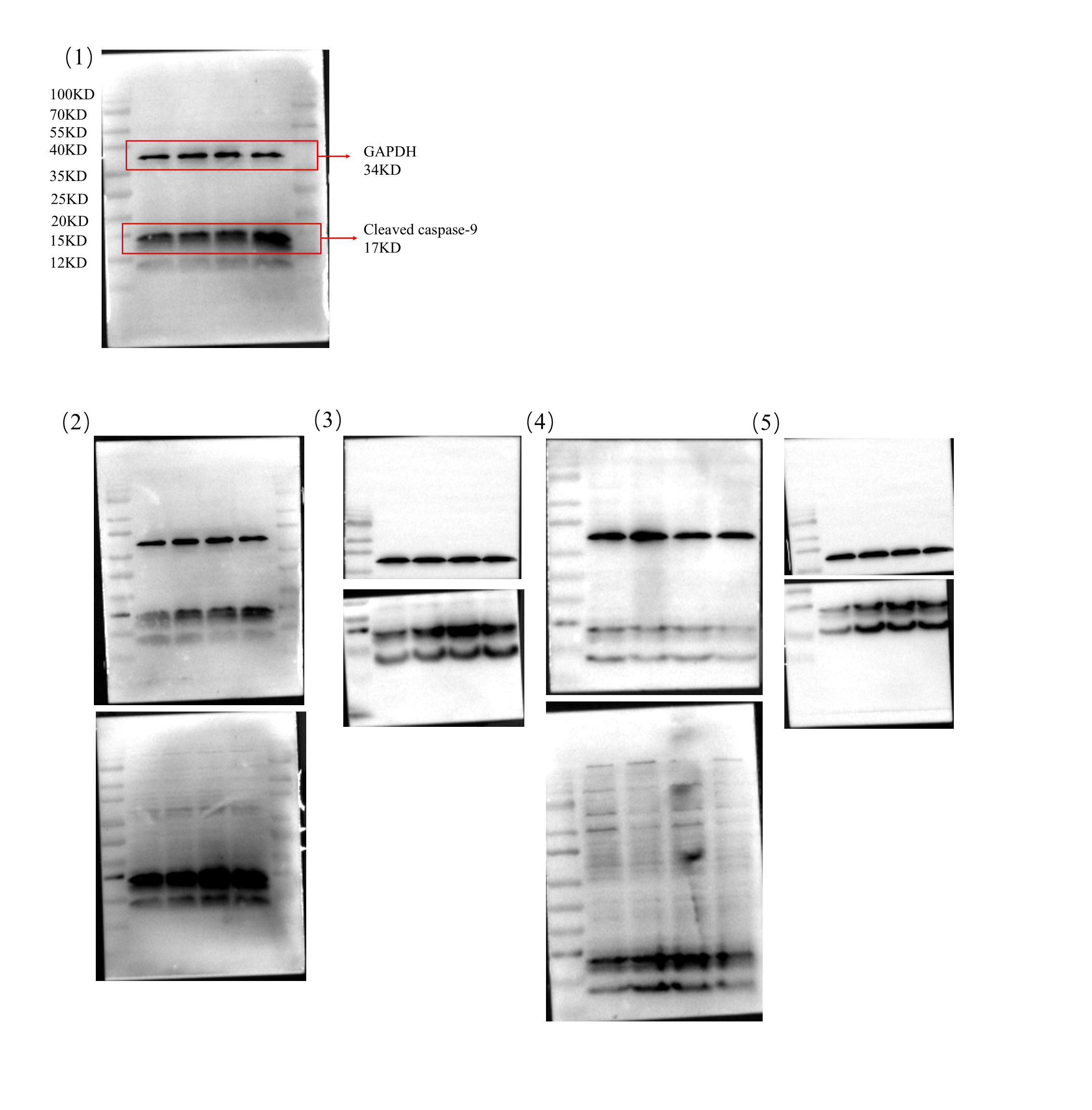


Expression of Cleaved caspase-9 in GH3 cells, 5 replicate experiments.


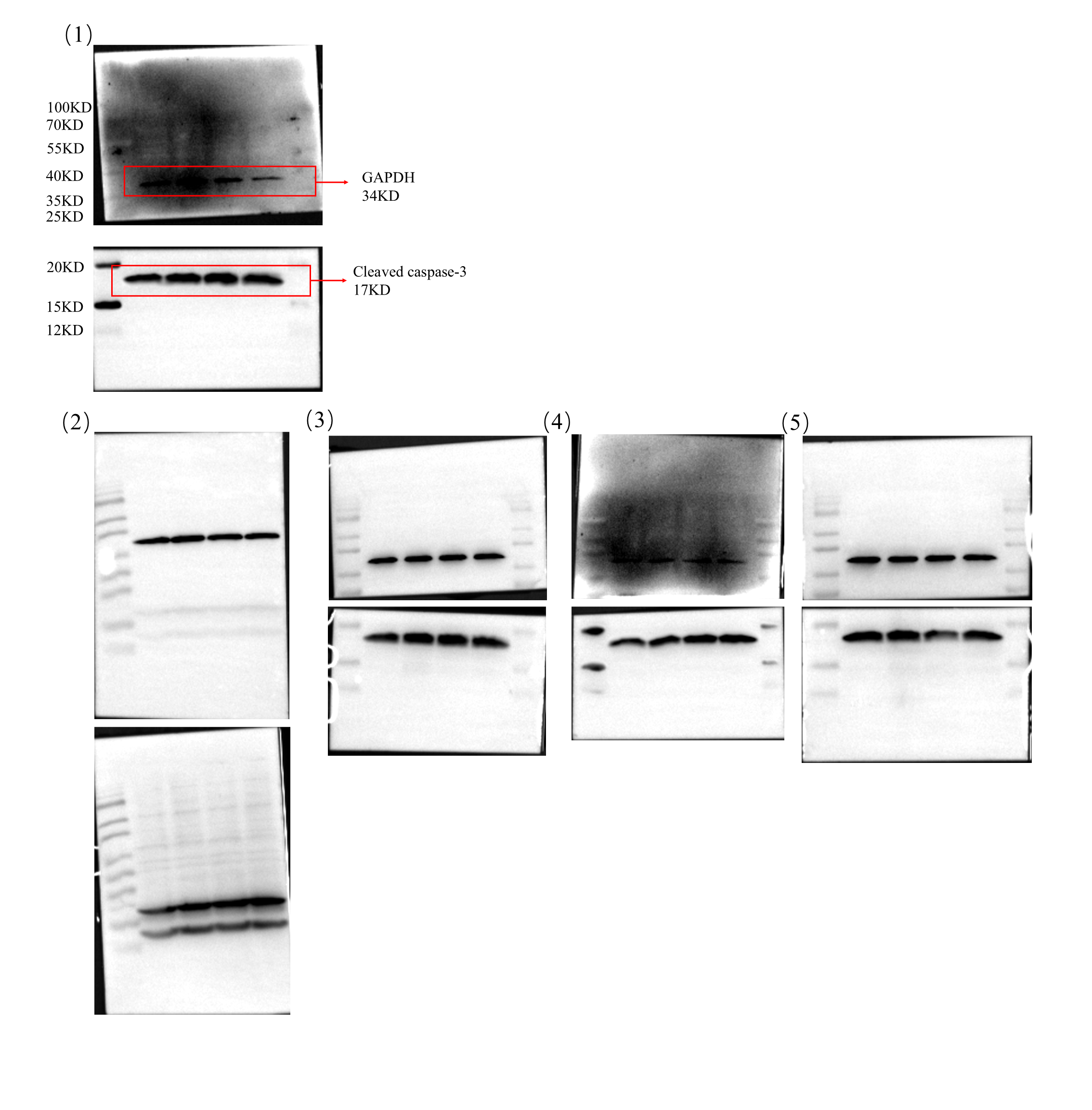


Expression of Cleaved caspase-3 in GH3 cells, 5 replicate experiments.


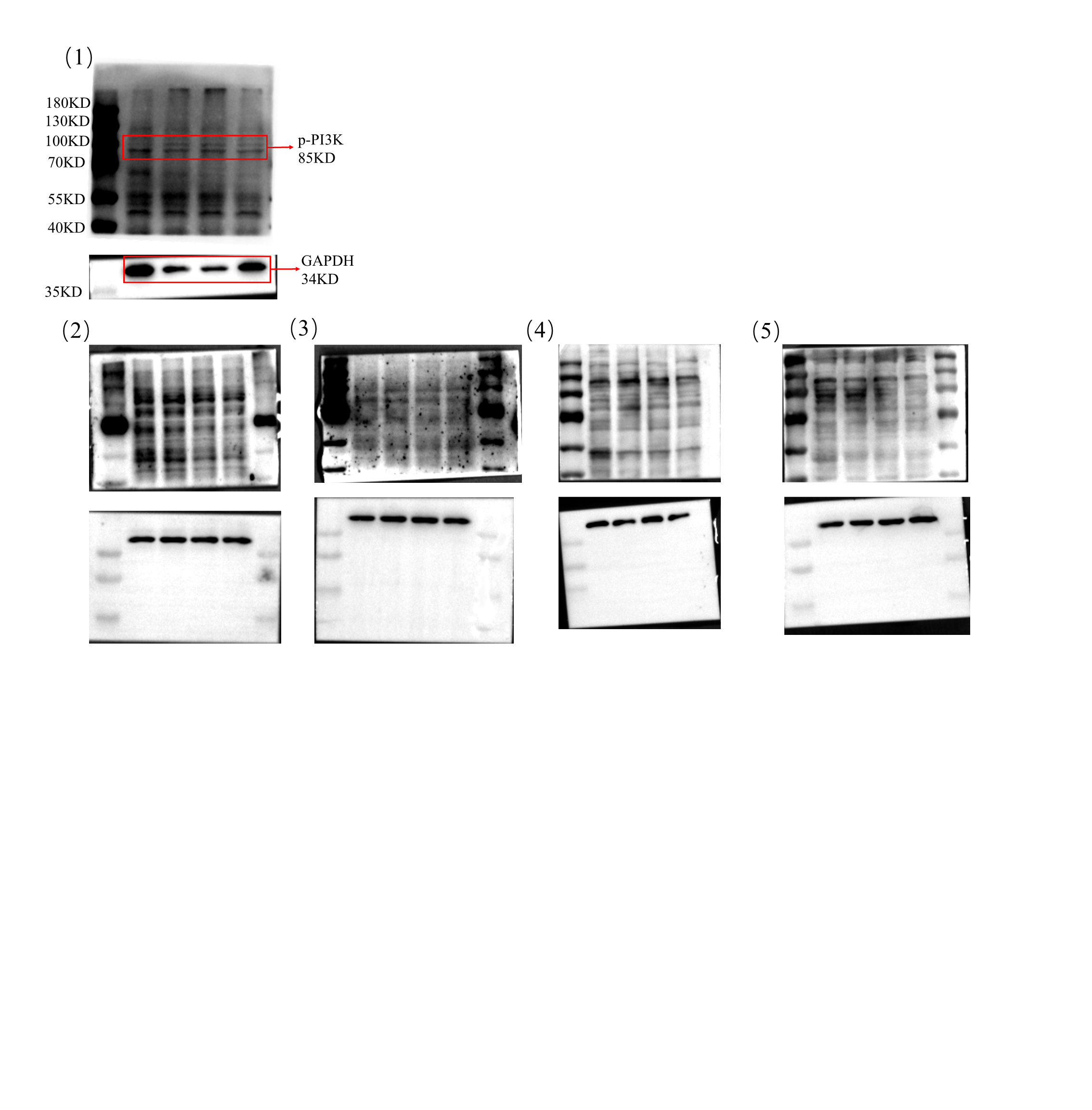


Expression of p-PI3K in MMQ cells, 5 replicate experiments.


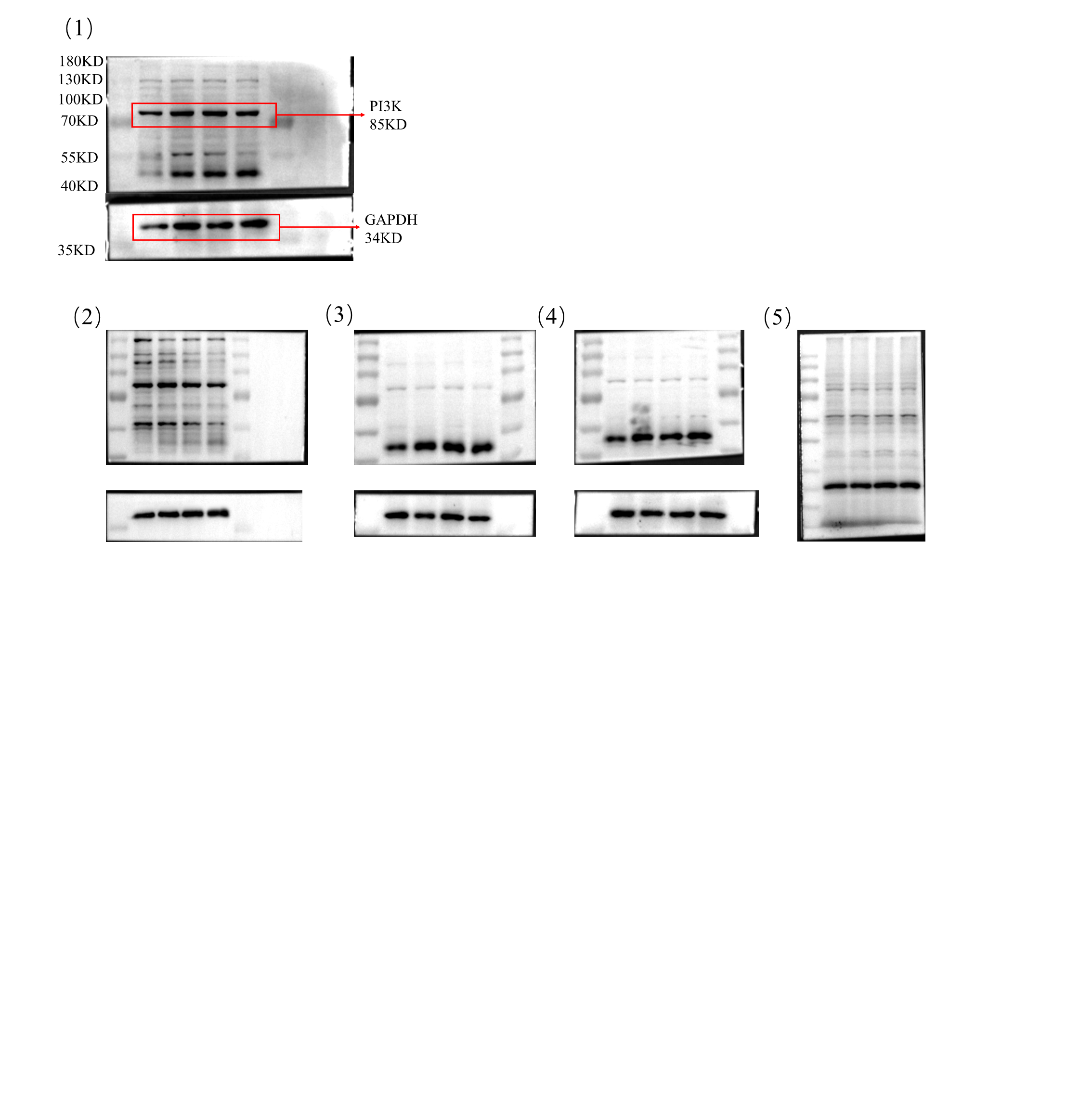


Expression of PI3K in MMQ cells, 5 replicate experiments.


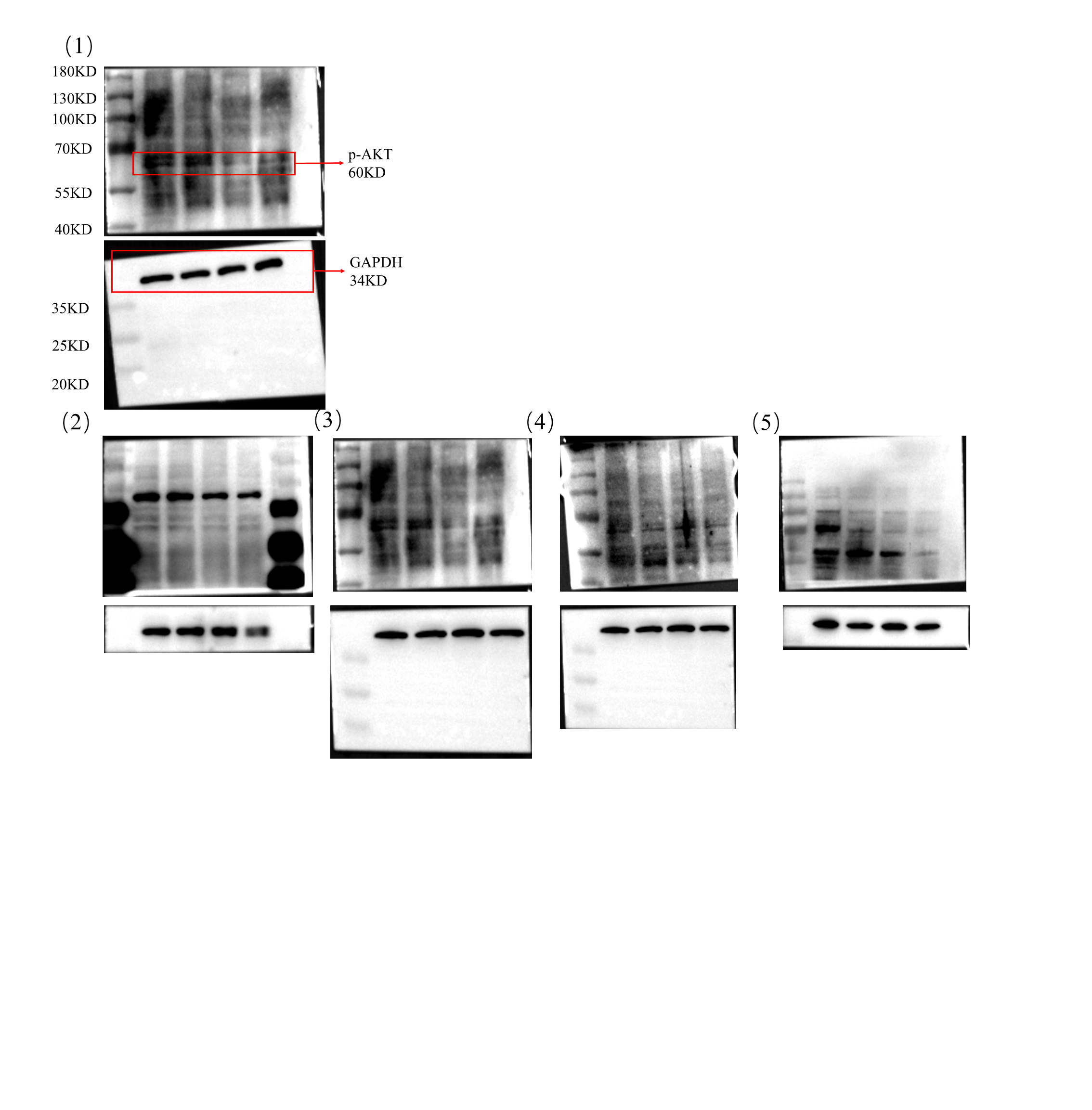


Expression of p-AKT in MMQ cells, 5 replicate experiments.


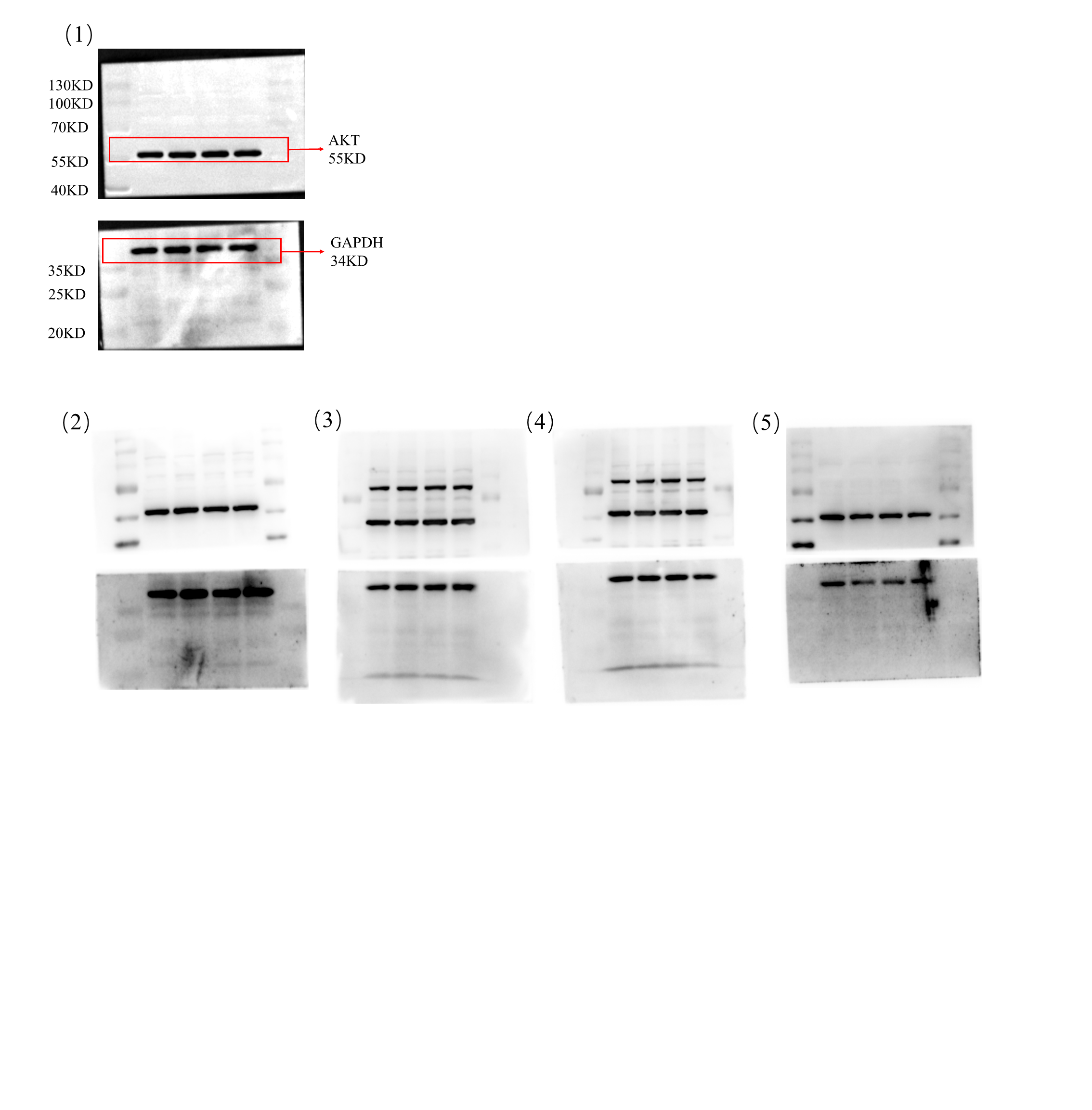


Expression of AKT in MMQ cells, 5 replicate experiments.


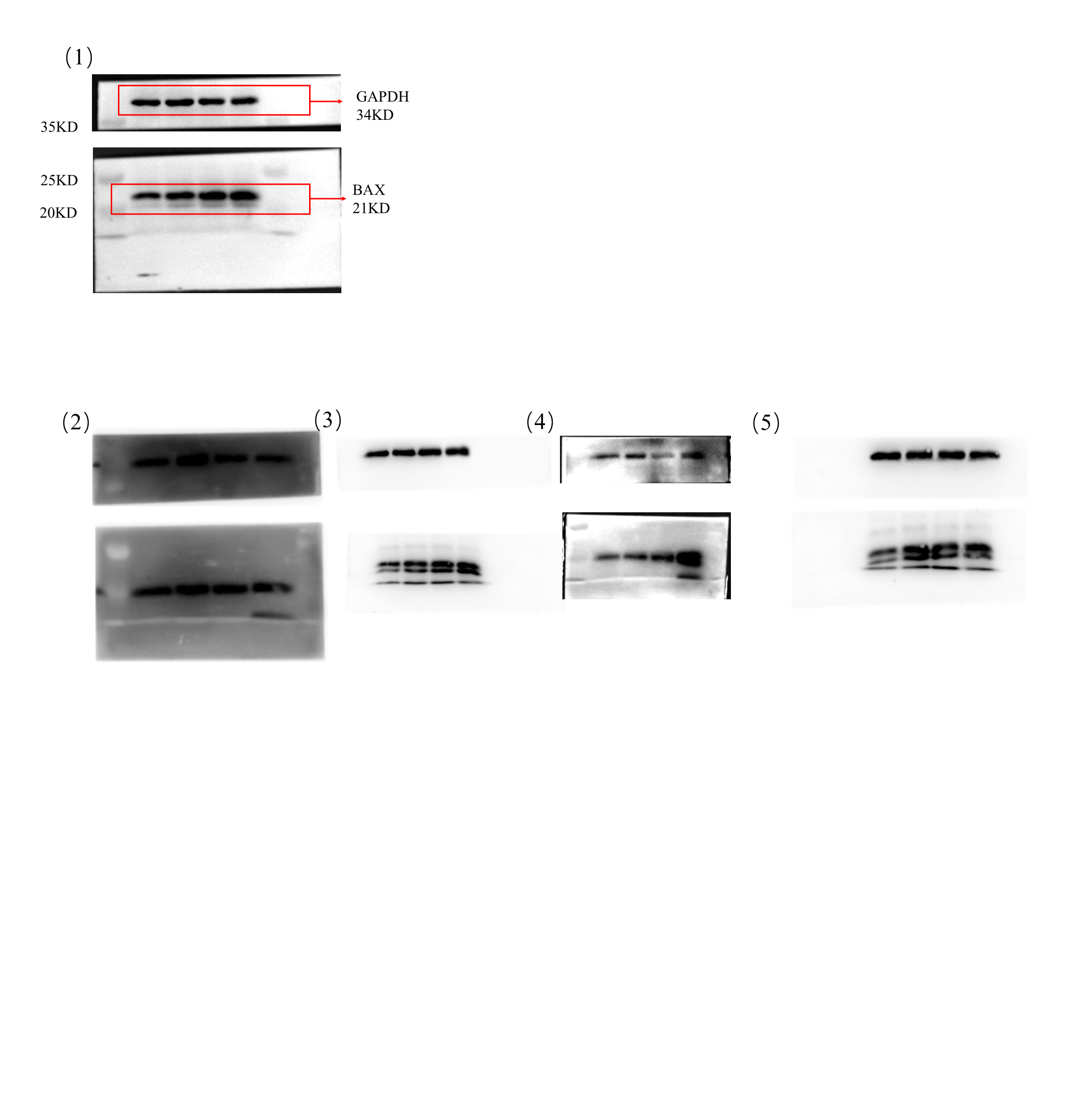


Expression of BAX in MMQ cells, 5 replicate experiments.


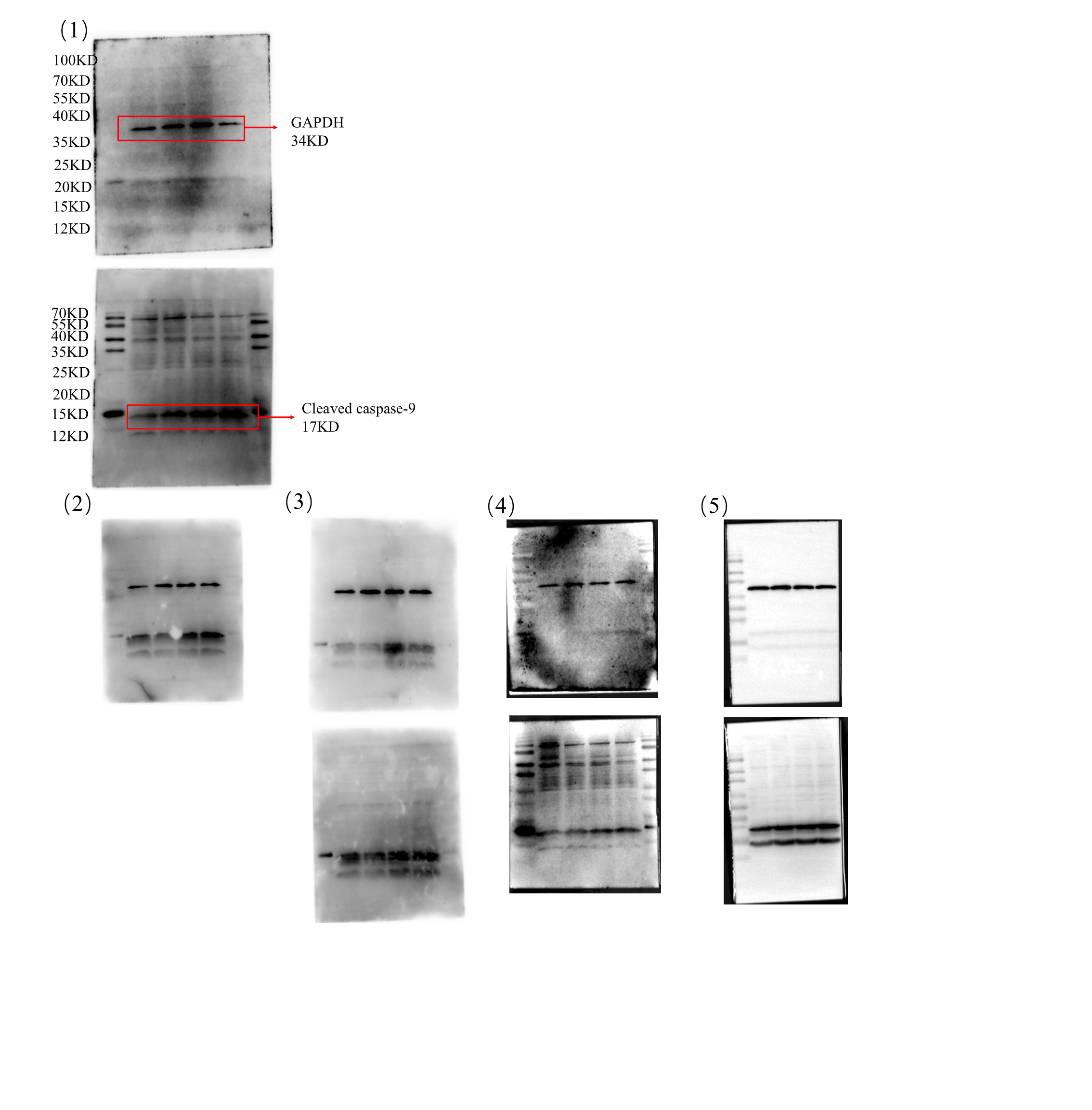


Expression of Cleaved caspase-9 in MMQ cells, 5 replicate experiments.


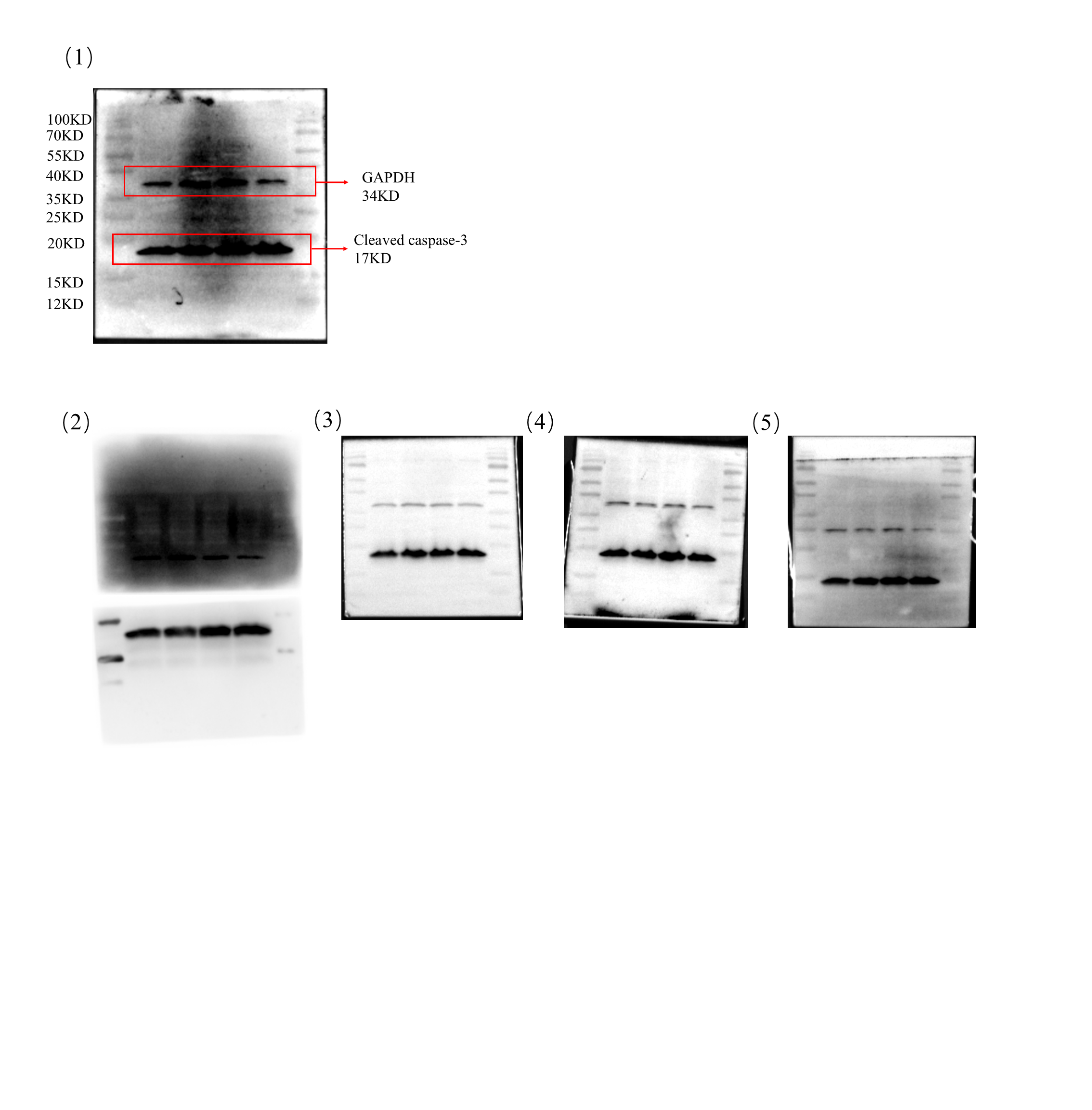


Expression of Cleaved caspase-3 in MMQ cells, 5 replicate experiments.
