# Supplementary material for: Wogonin inhibits the proliferation of prolactinoma through the PI3K/AKT signaling pathway
Source: Front Pharmacol. 2025 May 15;16:1546285. doi: 10.3389/fphar.2025.1546285 (PMC12119628; doi:10.3389/fphar.2025.1546285)
Supplement: Supplementary file 2 [file DataSheet1.docx]

***Supplementary Material***

**Supplementary Figures**


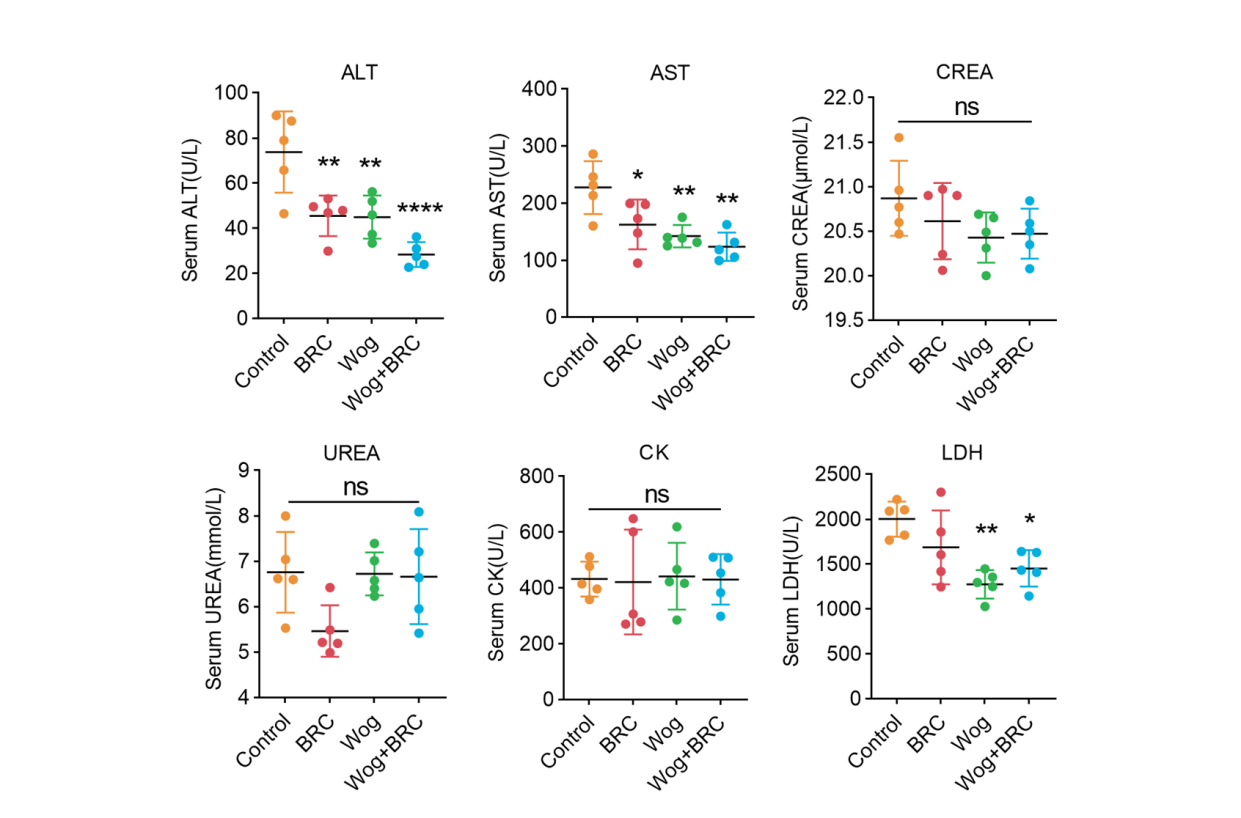


**Supplementary Figure 1.** Statistical graphs of serum biochemical analysis: Alanine transaminase (ALT); Aspartate aminotransferase (AST); Creatinine (CREA); Urea (UREA); Creatine kinase (CK); Lactate dehydrogenase (LDH). (**p < 0.05, **p < 0.01, ****p < 0.0001, all * indicate comparisons with the control group, ns means no Significance.*)


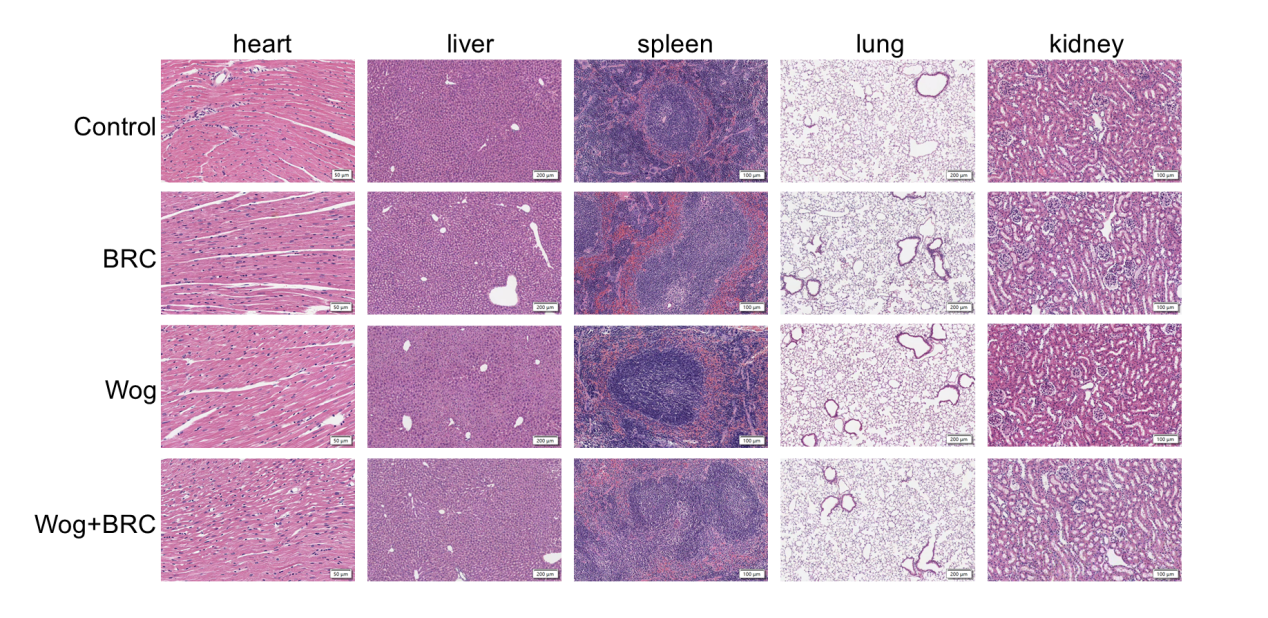


**Supplementary Figure 2.** The representative images of HE staining of the heart, liver, spleen, lungs and kidneys.
